# Supplementary figures and images for: The NAD+ precursor NMN activates dSarm to trigger axon degeneration in Drosophila
Source: eLife. 2022 Dec 23;11:e80245. doi: 10.7554/eLife.80245 (PMC9788811; doi:10.7554/eLife.80245)

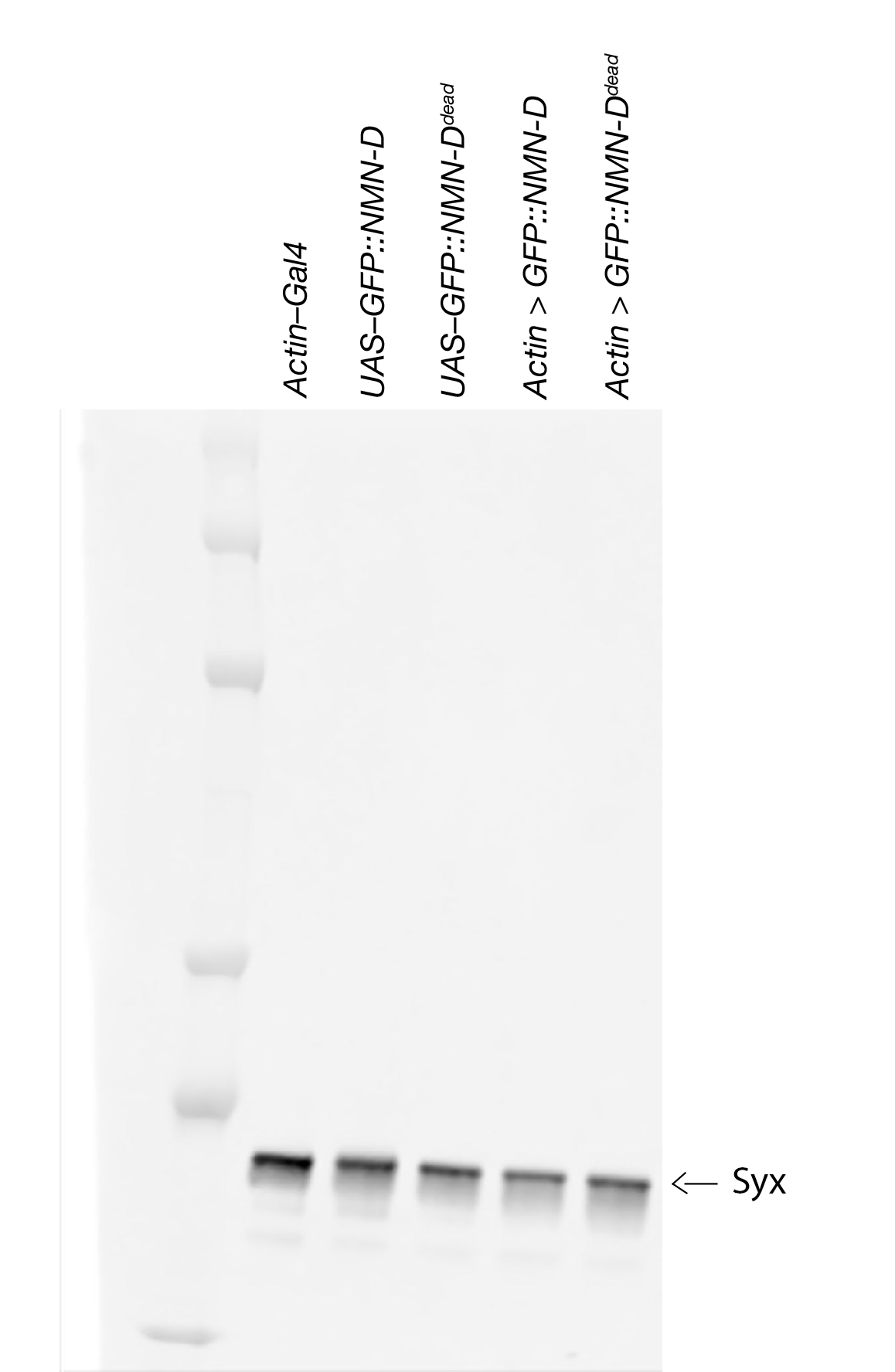

Supplement: Figure 1—source data 1. — (A) Raw unedited western blots and uncropped blots with relevant bands clearly labeled. (C) Raw data of quantified preserved severed axons. [file elife-80245-fig1-data1.zip › Figure 1/1A Western/syntaxin + info.png]

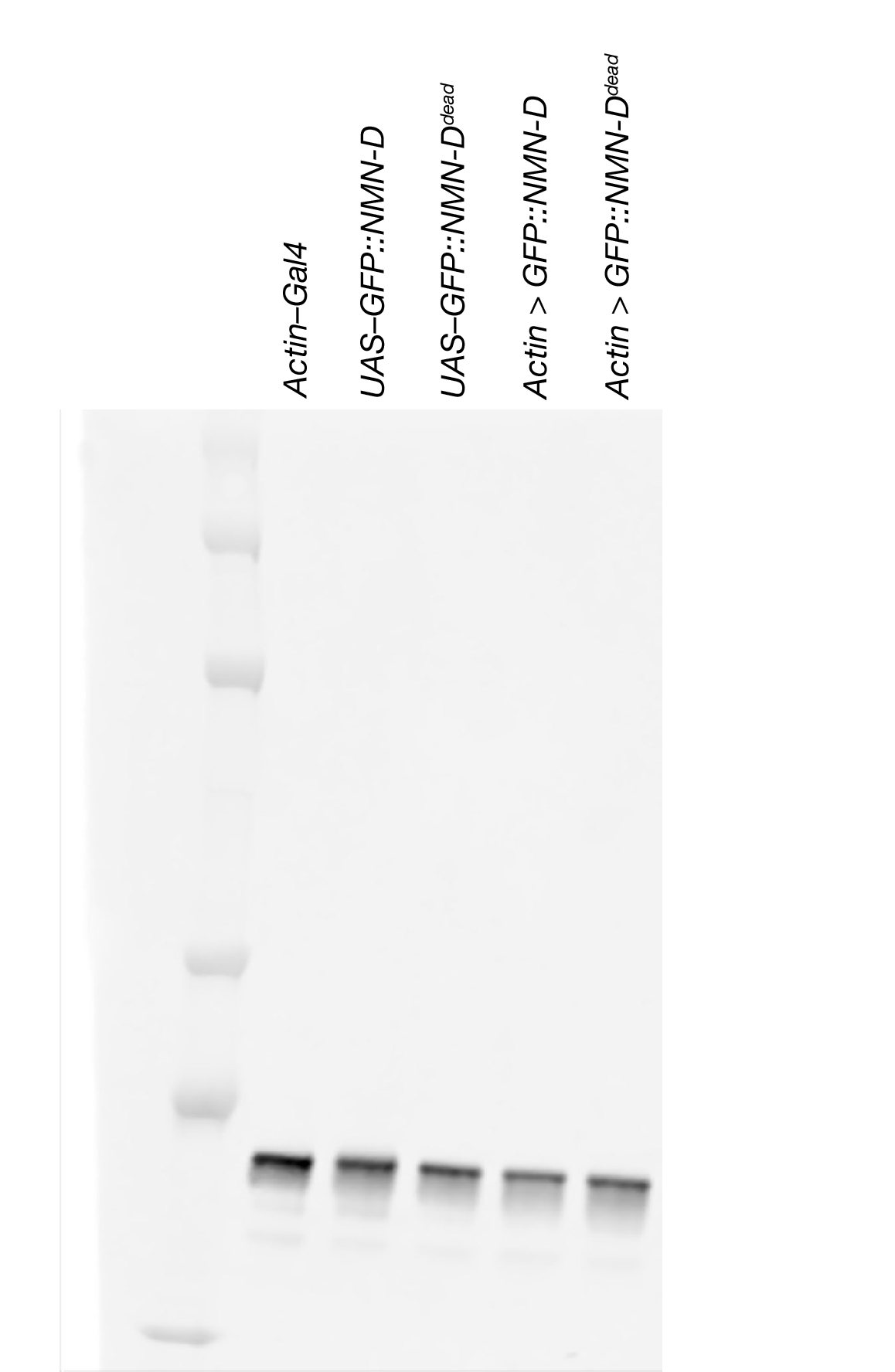

Supplement: Figure 1—source data 1. — (A) Raw unedited western blots and uncropped blots with relevant bands clearly labeled. (C) Raw data of quantified preserved severed axons. [file elife-80245-fig1-data1.zip › Figure 1/1A Western/syntaxin + info.jpg]

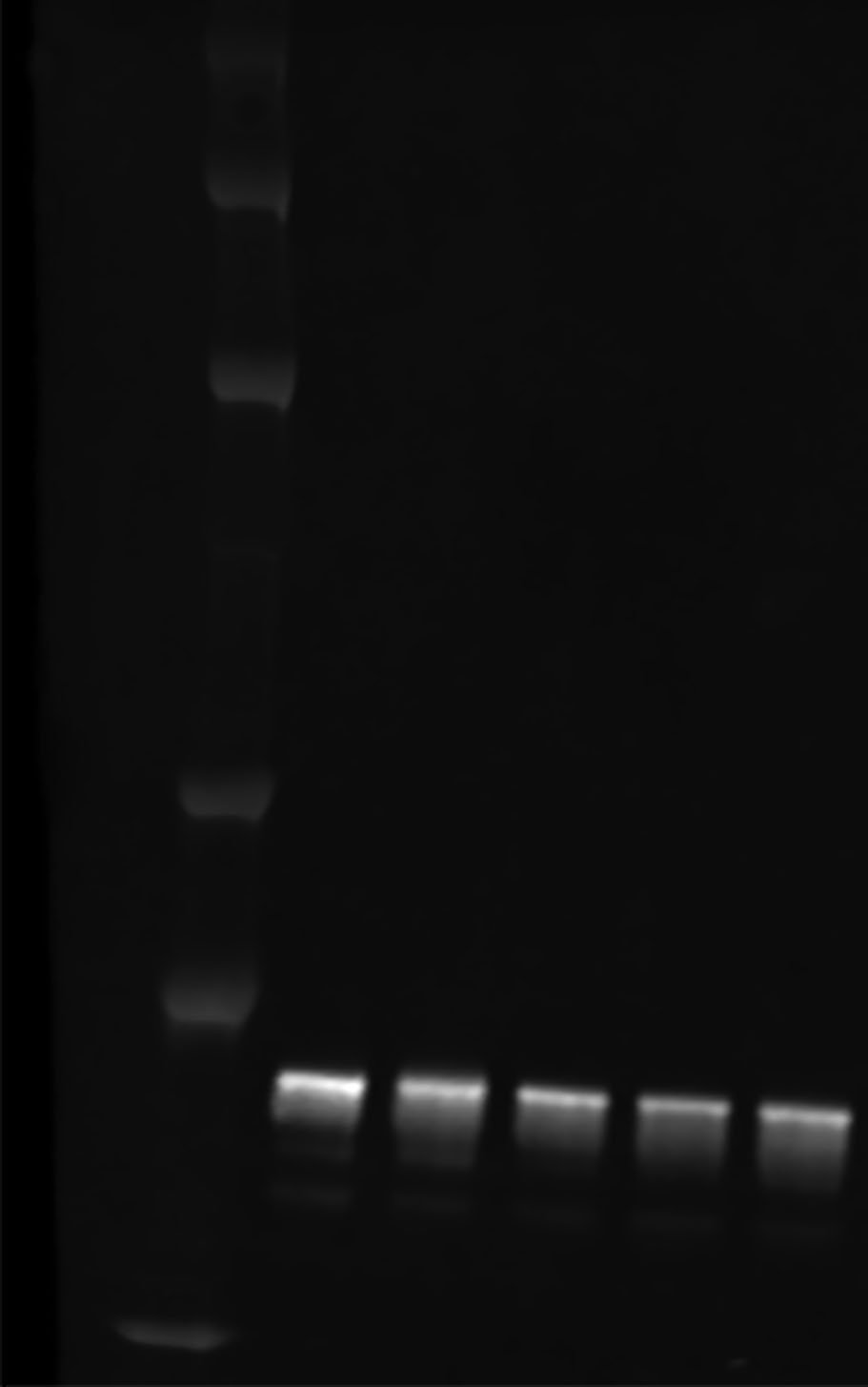

Supplement: Figure 1—source data 1. — (A) Raw unedited western blots and uncropped blots with relevant bands clearly labeled. (C) Raw data of quantified preserved severed axons. [file elife-80245-fig1-data1.zip › Figure 1/1A Western/syntaxin raw.jpg]

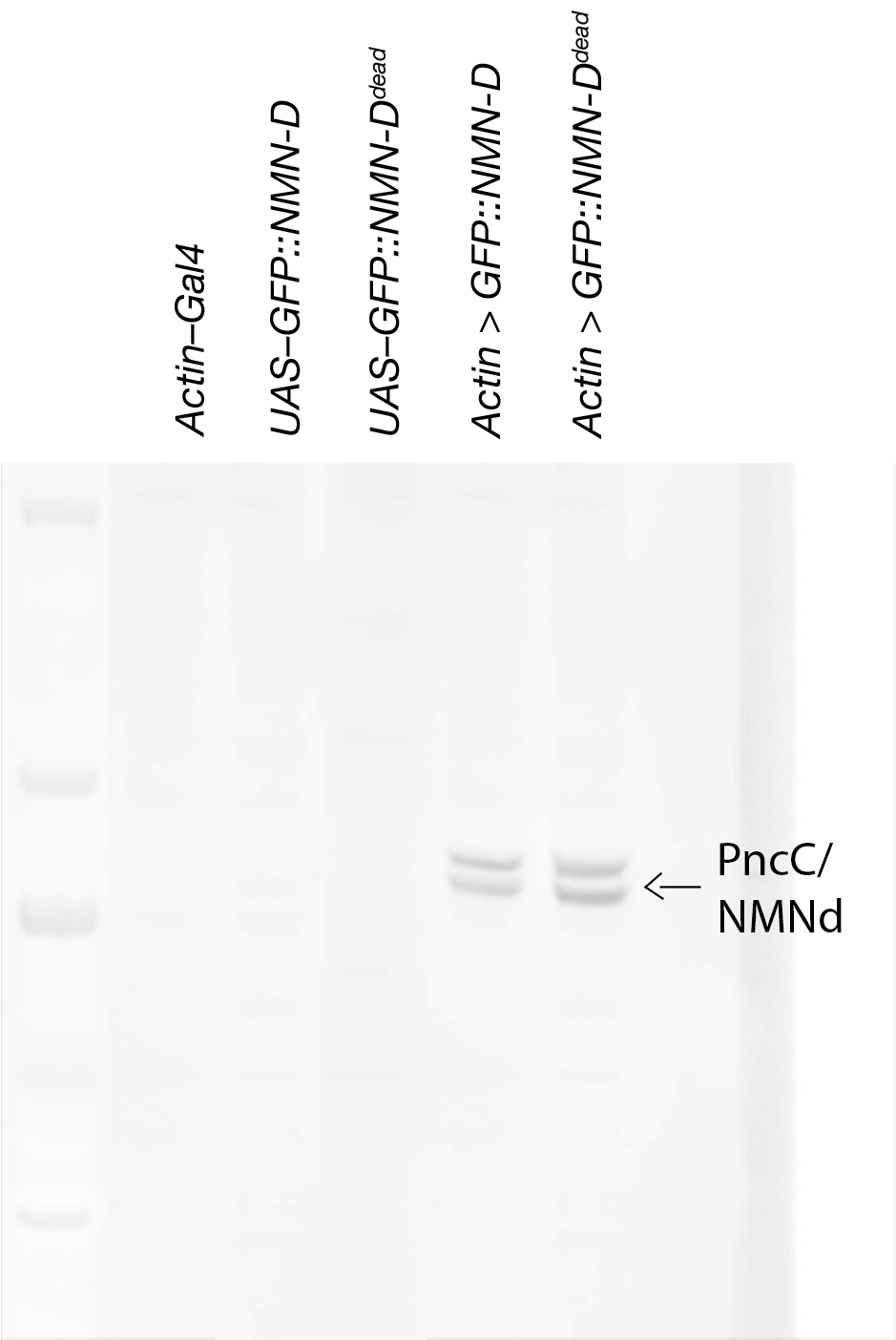

Supplement: Figure 1—source data 1. — (A) Raw unedited western blots and uncropped blots with relevant bands clearly labeled. (C) Raw data of quantified preserved severed axons. [file elife-80245-fig1-data1.zip › Figure 1/1A Western/red channel + info.png]

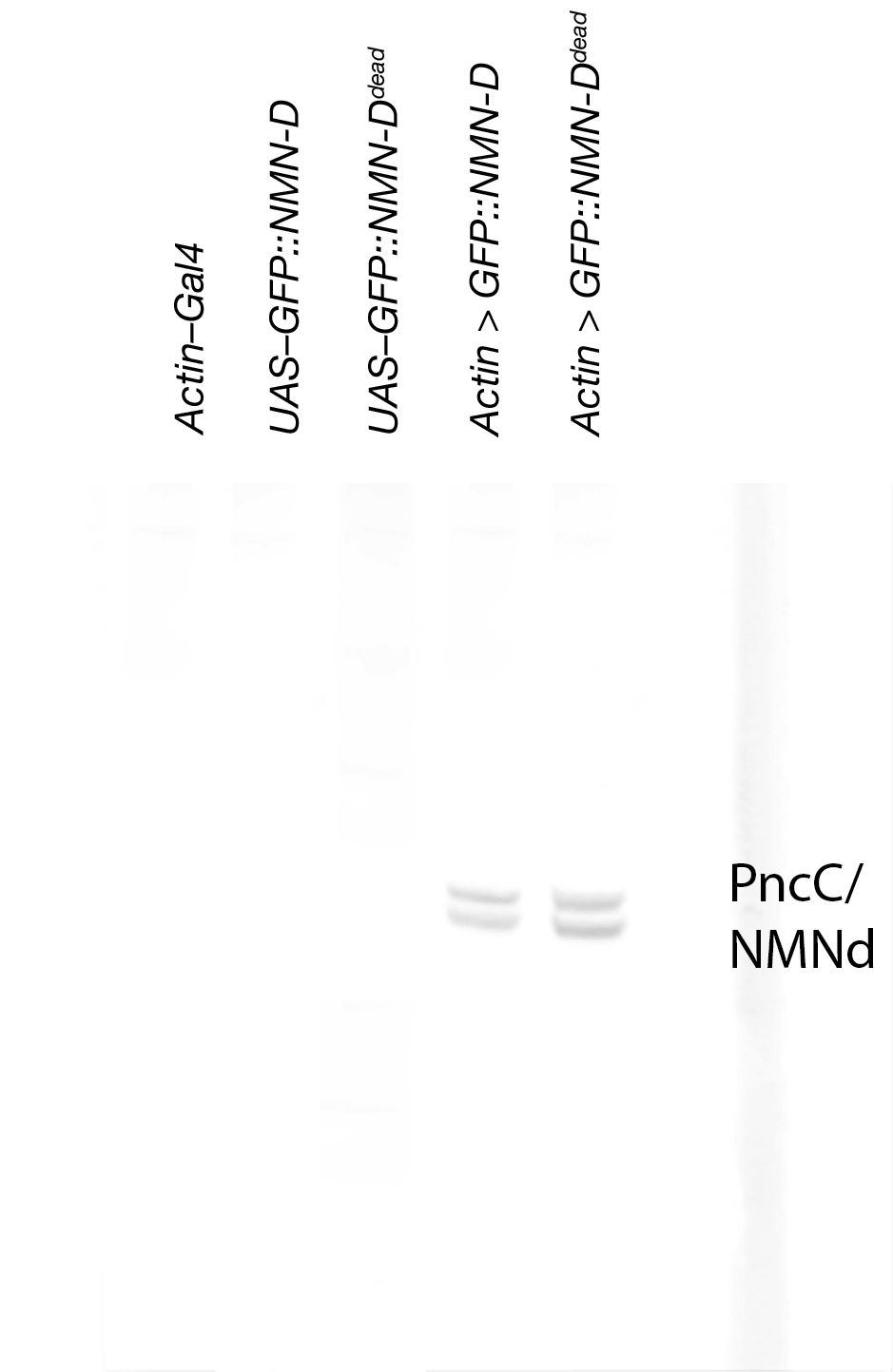

Supplement: Figure 1—source data 1. — (A) Raw unedited western blots and uncropped blots with relevant bands clearly labeled. (C) Raw data of quantified preserved severed axons. [file elife-80245-fig1-data1.zip › Figure 1/1A Western/green channel +info.png]

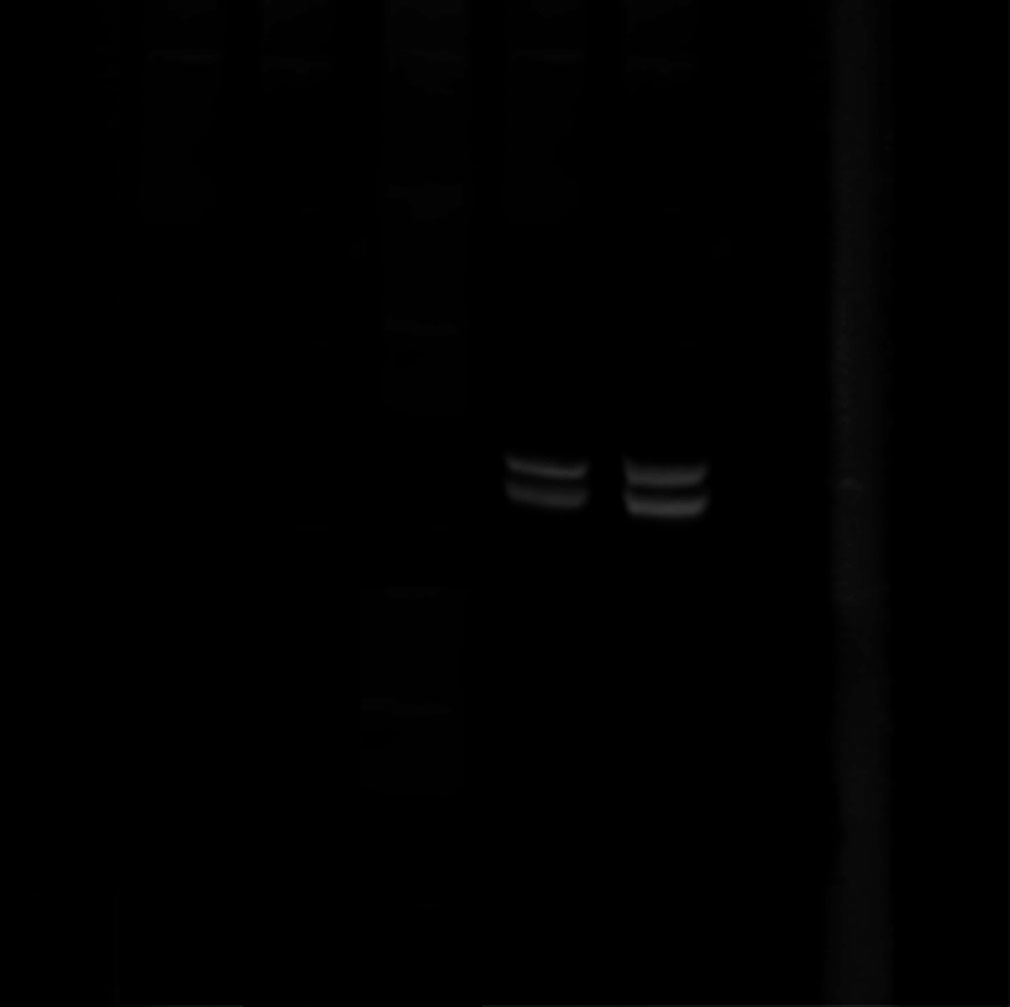

Supplement: Figure 1—source data 1. — (A) Raw unedited western blots and uncropped blots with relevant bands clearly labeled. (C) Raw data of quantified preserved severed axons. [file elife-80245-fig1-data1.zip › Figure 1/1A Western/green channel raw.jpg]

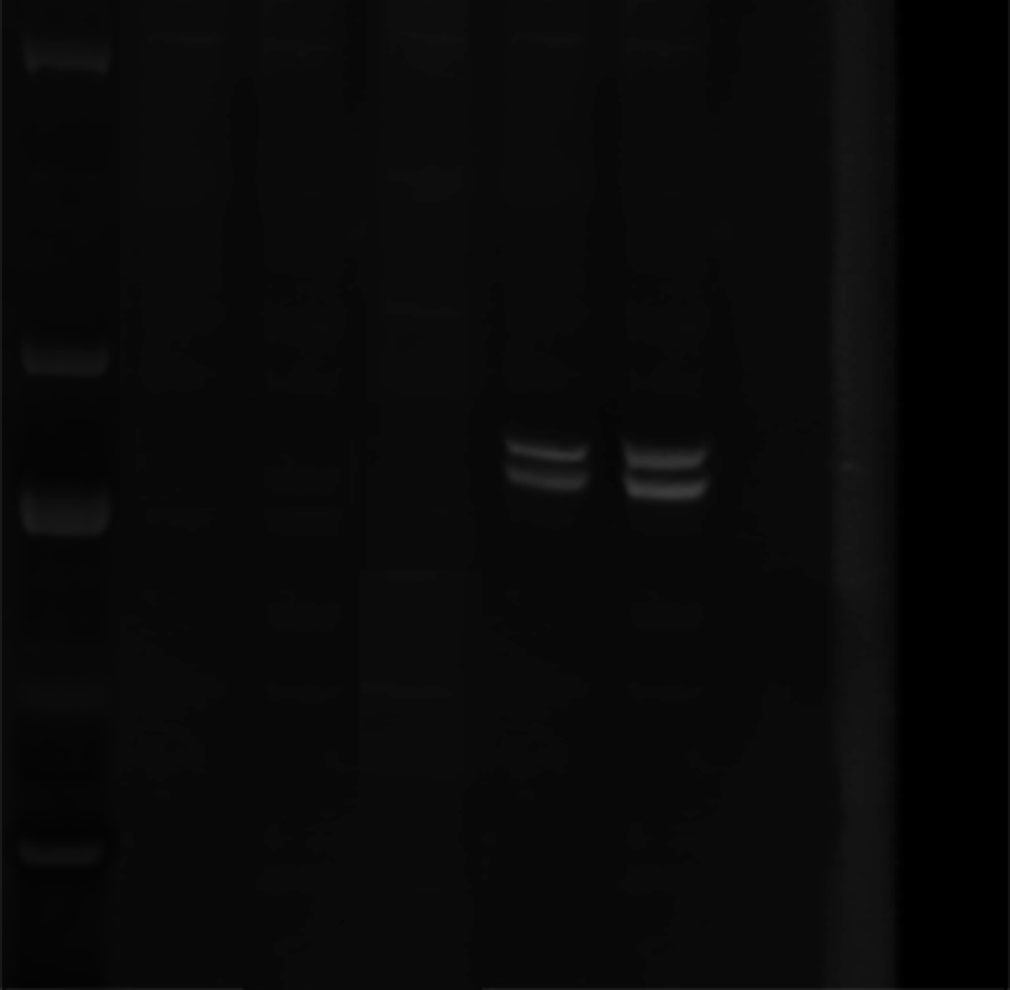

Supplement: Figure 1—source data 1. — (A) Raw unedited western blots and uncropped blots with relevant bands clearly labeled. (C) Raw data of quantified preserved severed axons. [file elife-80245-fig1-data1.zip › Figure 1/1A Western/red channel raw.jpg]

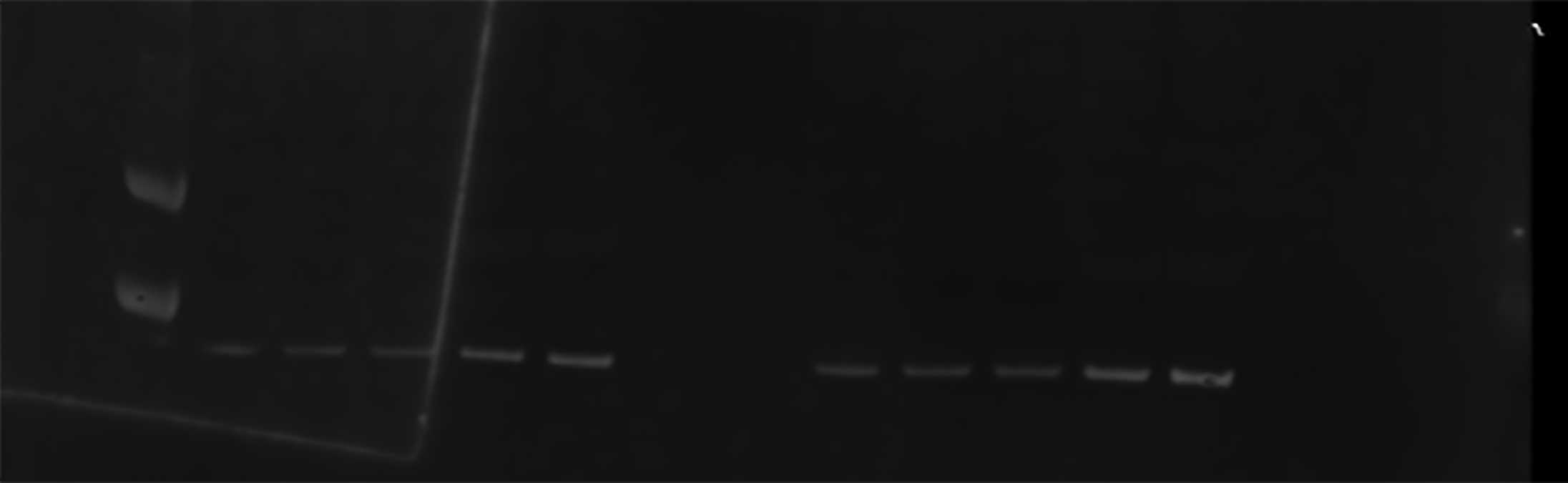

Supplement: Figure 1—figure supplement 2—source data 1. — (A) Raw unedited western blots and uncropped blots with relevant bands clearly labeled. (B) Raw unedited western blots and uncropped blots with relevant bands clearly labeled. (C) Raw data of Western blot quantification. [file elife-80245-fig1-figsupp2-data1.zip › Figure 1 - figure supplement 2/1 S2A Western/syx raw.jpg]

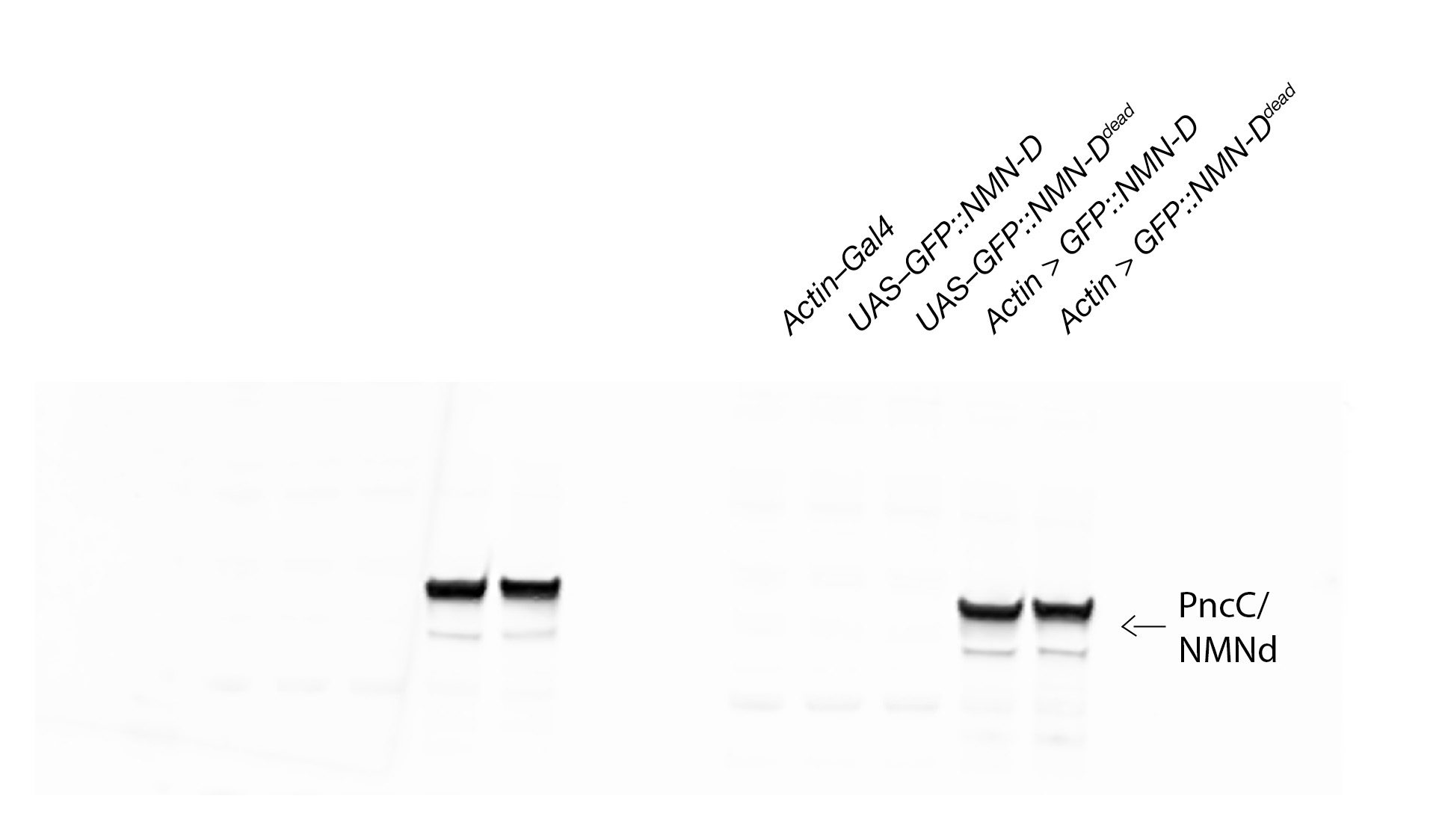

Supplement: Figure 1—figure supplement 2—source data 1. — (A) Raw unedited western blots and uncropped blots with relevant bands clearly labeled. (B) Raw unedited western blots and uncropped blots with relevant bands clearly labeled. (C) Raw data of Western blot quantification. [file elife-80245-fig1-figsupp2-data1.zip › Figure 1 - figure supplement 2/1 S2A Western/pncc + info.jpg]

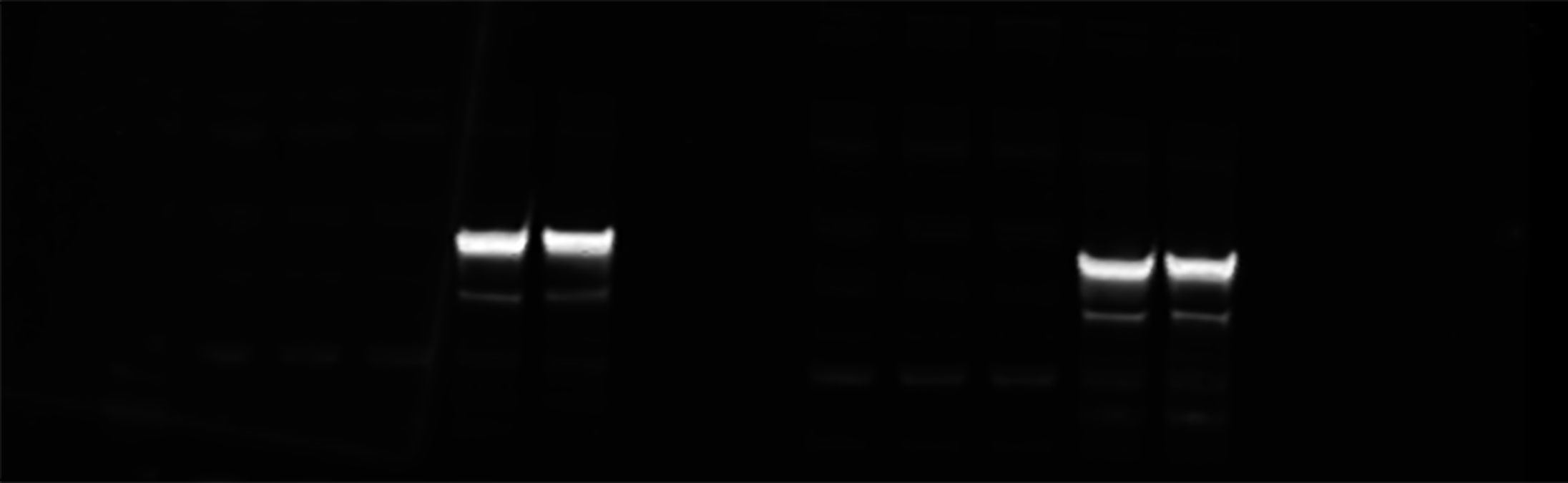

Supplement: Figure 1—figure supplement 2—source data 1. — (A) Raw unedited western blots and uncropped blots with relevant bands clearly labeled. (B) Raw unedited western blots and uncropped blots with relevant bands clearly labeled. (C) Raw data of Western blot quantification. [file elife-80245-fig1-figsupp2-data1.zip › Figure 1 - figure supplement 2/1 S2A Western/pncc raw.jpg]

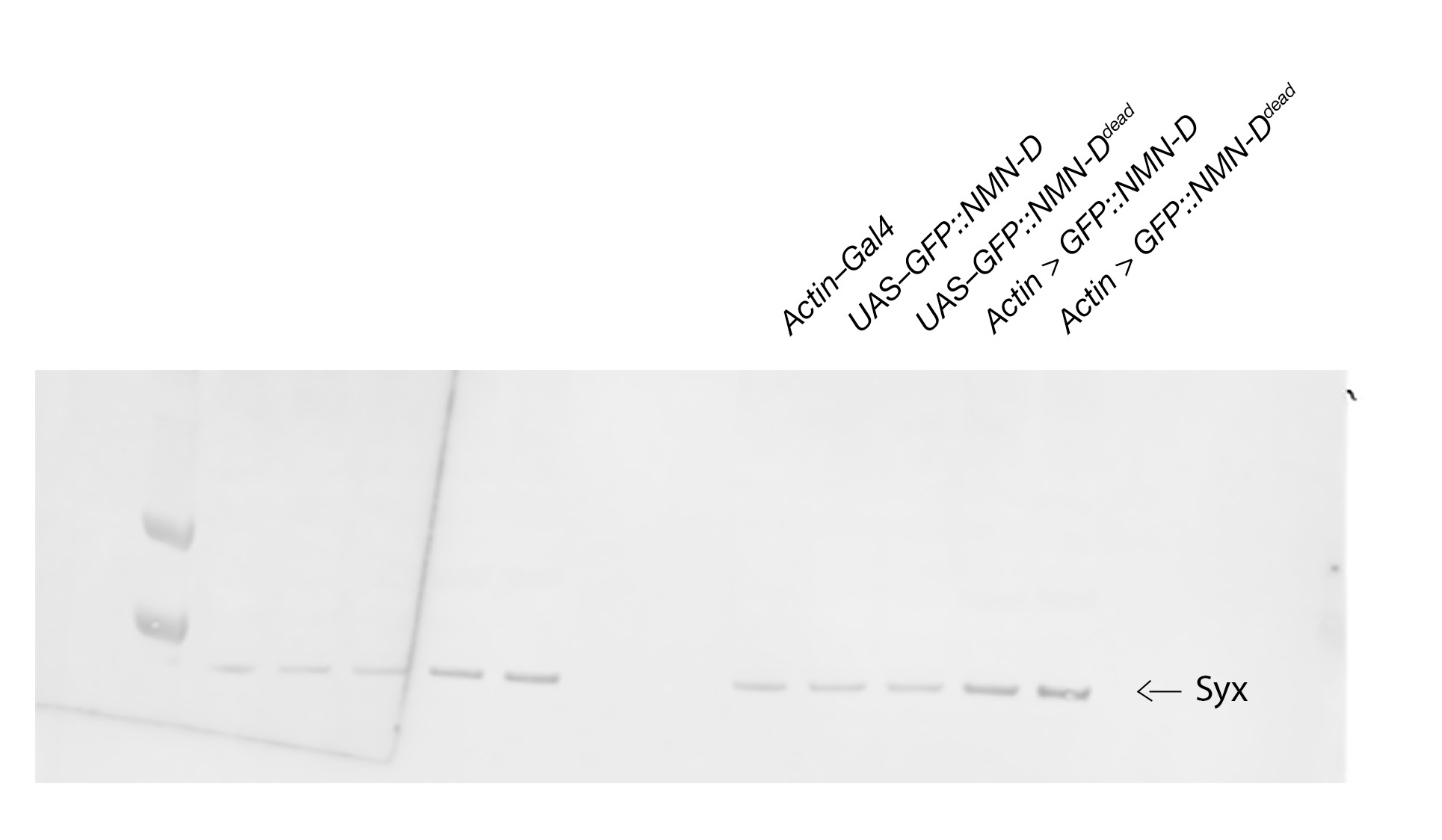

Supplement: Figure 1—figure supplement 2—source data 1. — (A) Raw unedited western blots and uncropped blots with relevant bands clearly labeled. (B) Raw unedited western blots and uncropped blots with relevant bands clearly labeled. (C) Raw data of Western blot quantification. [file elife-80245-fig1-figsupp2-data1.zip › Figure 1 - figure supplement 2/1 S2A Western/syx + info.jpg]

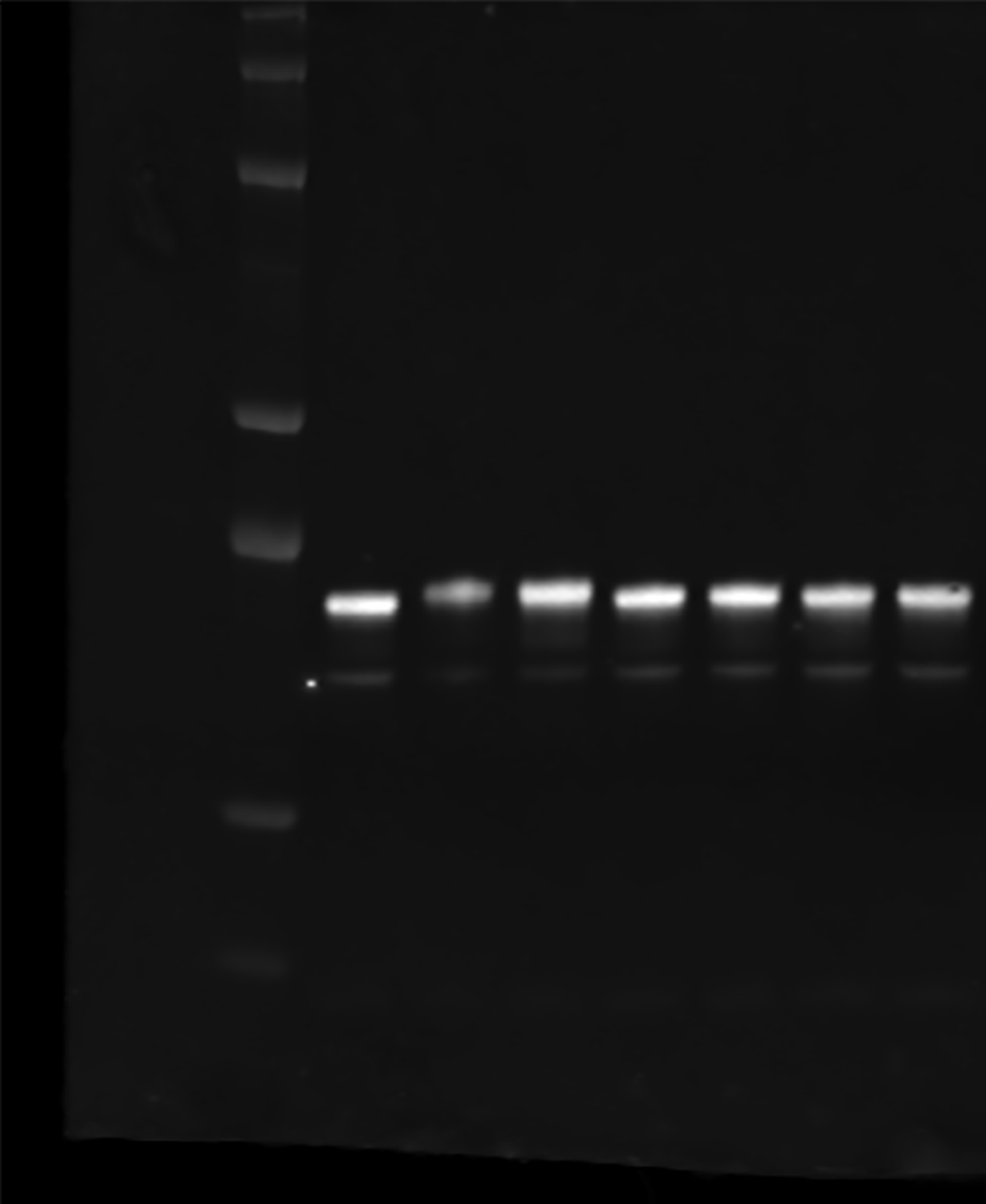

Supplement: Figure 1—figure supplement 2—source data 1. — (A) Raw unedited western blots and uncropped blots with relevant bands clearly labeled. (B) Raw unedited western blots and uncropped blots with relevant bands clearly labeled. (C) Raw data of Western blot quantification. [file elife-80245-fig1-figsupp2-data1.zip › Figure 1 - figure supplement 2/1 S2B Western/syx raw.jpg]

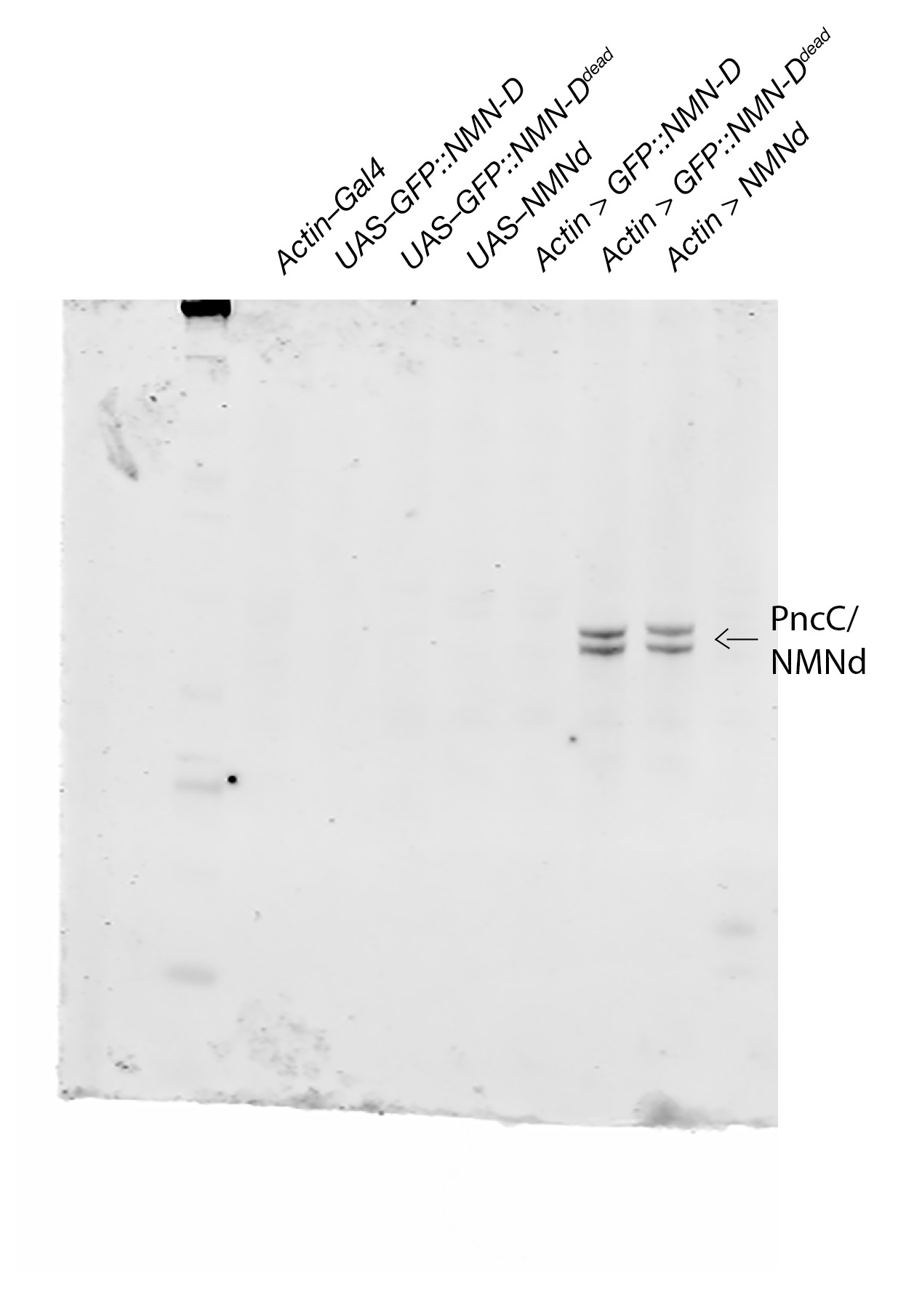

Supplement: Figure 1—figure supplement 2—source data 1. — (A) Raw unedited western blots and uncropped blots with relevant bands clearly labeled. (B) Raw unedited western blots and uncropped blots with relevant bands clearly labeled. (C) Raw data of Western blot quantification. [file elife-80245-fig1-figsupp2-data1.zip › Figure 1 - figure supplement 2/1 S2B Western/pncc + info.jpg]

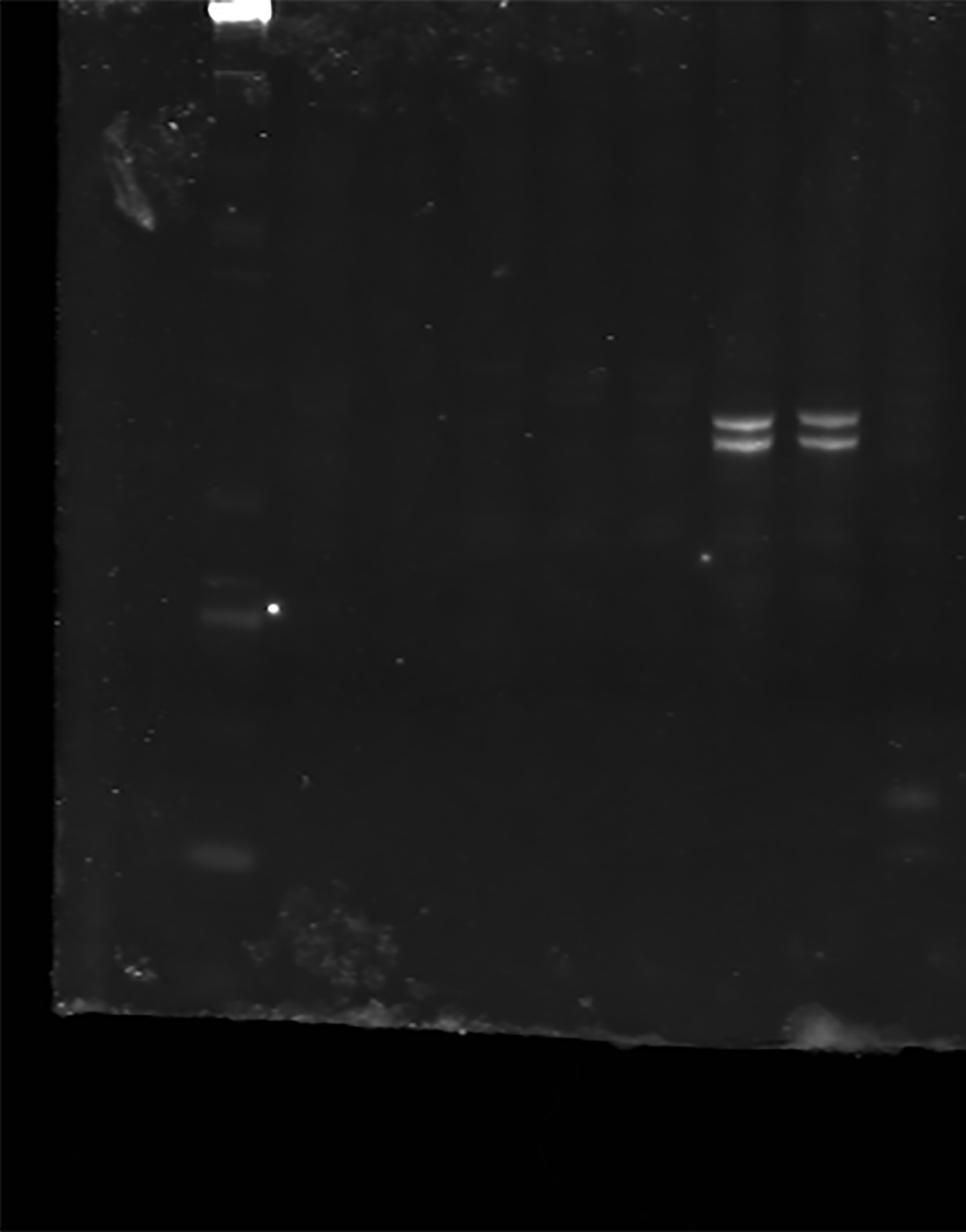

Supplement: Figure 1—figure supplement 2—source data 1. — (A) Raw unedited western blots and uncropped blots with relevant bands clearly labeled. (B) Raw unedited western blots and uncropped blots with relevant bands clearly labeled. (C) Raw data of Western blot quantification. [file elife-80245-fig1-figsupp2-data1.zip › Figure 1 - figure supplement 2/1 S2B Western/pncc raw.jpg]

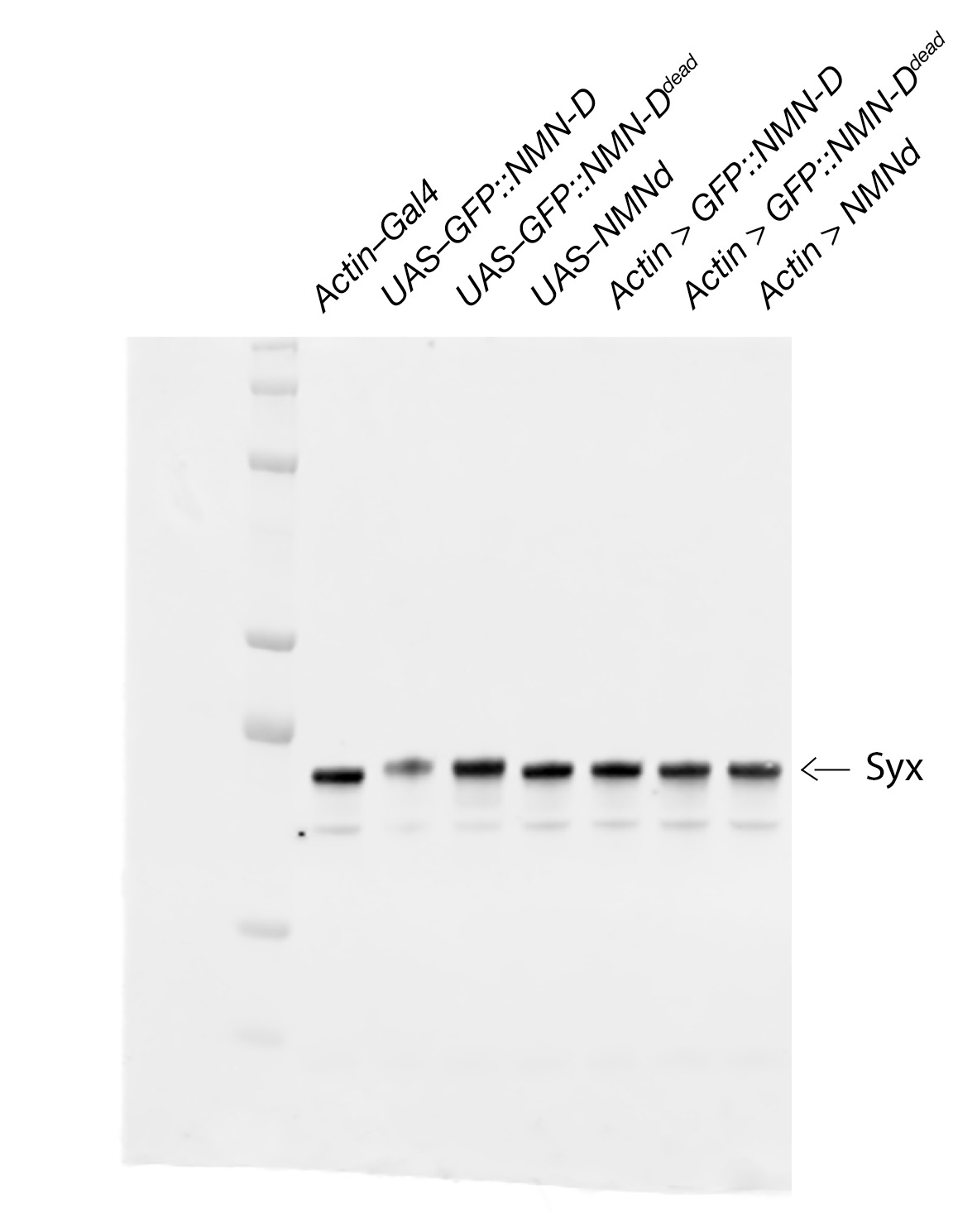

Supplement: Figure 1—figure supplement 2—source data 1. — (A) Raw unedited western blots and uncropped blots with relevant bands clearly labeled. (B) Raw unedited western blots and uncropped blots with relevant bands clearly labeled. (C) Raw data of Western blot quantification. [file elife-80245-fig1-figsupp2-data1.zip › Figure 1 - figure supplement 2/1 S2B Western/syx + info.jpg]

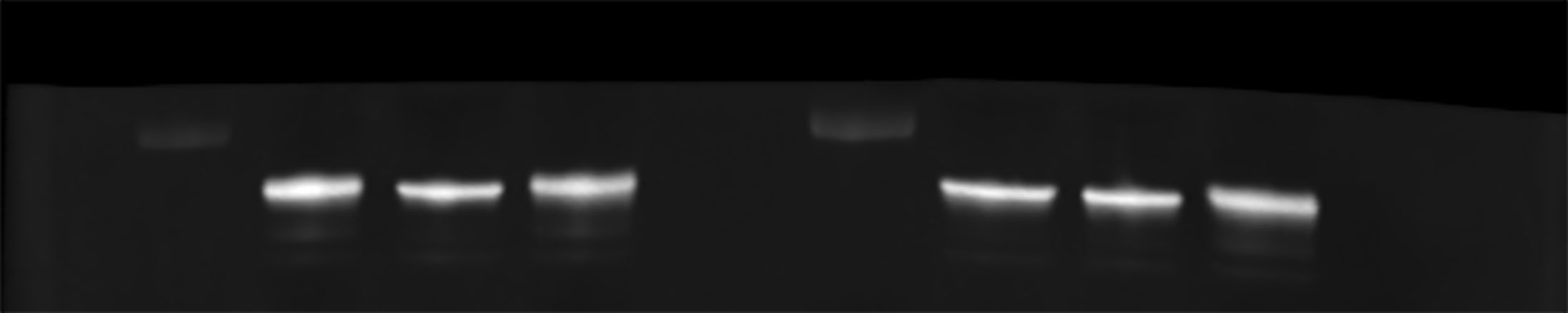

Supplement: Figure 4—source data 1. — (B) Raw unedited Western blots and uncropped blots with relevant bands clearly labeled. (C) Raw data of metabolomic measurement. (E) Raw data of quantified preserved severed axons. (F) Raw data of quantified preserved severed axons. (G) Raw data of quantified preserved severed axons. [file elife-80245-fig4-data1.zip › Figure 4/4B wb/syx raw.jpg]

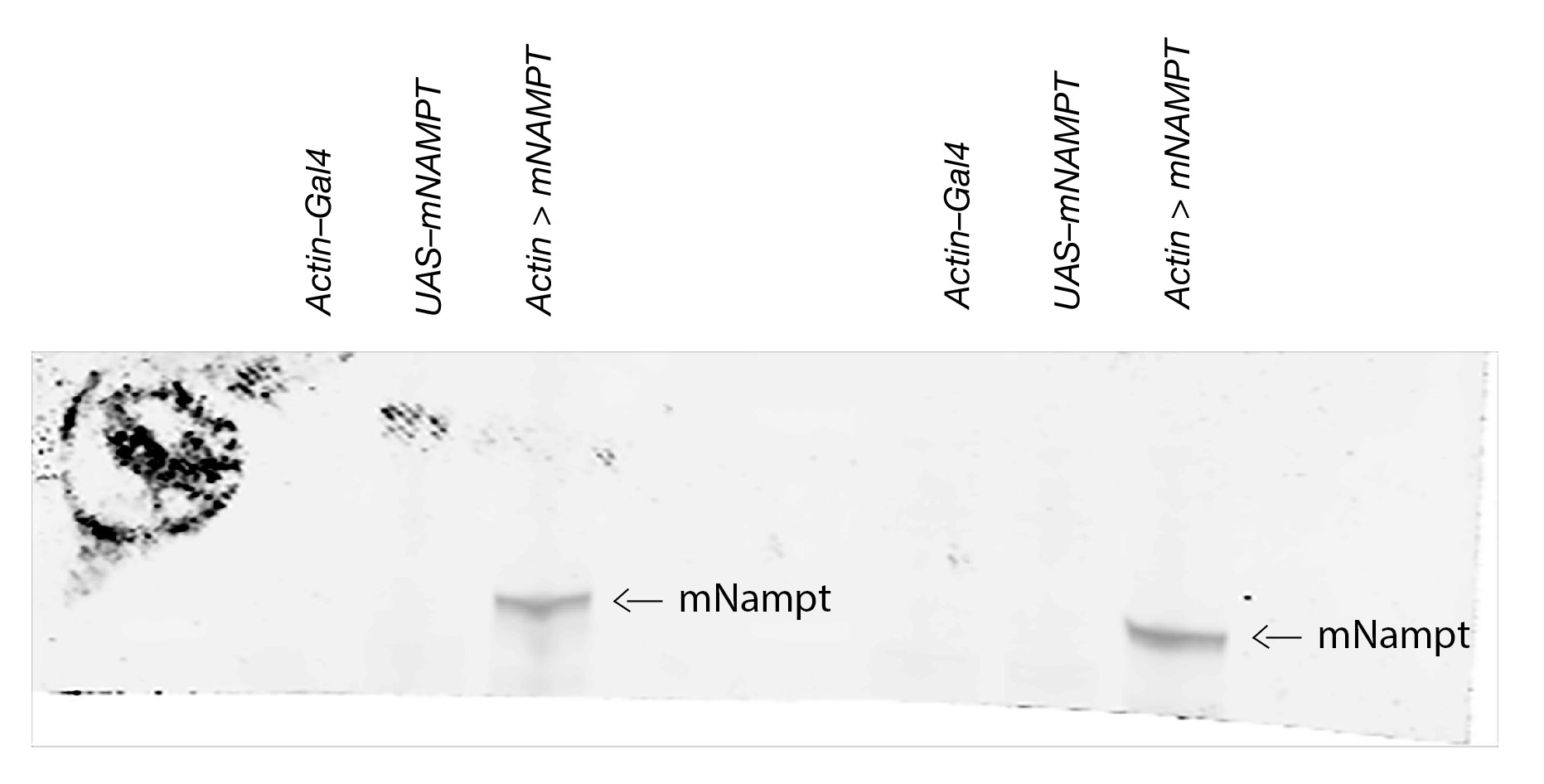

Supplement: Figure 4—source data 1. — (B) Raw unedited Western blots and uncropped blots with relevant bands clearly labeled. (C) Raw data of metabolomic measurement. (E) Raw data of quantified preserved severed axons. (F) Raw data of quantified preserved severed axons. (G) Raw data of quantified preserved severed axons. [file elife-80245-fig4-data1.zip › Figure 4/4B wb/mnampt + info.jpg]

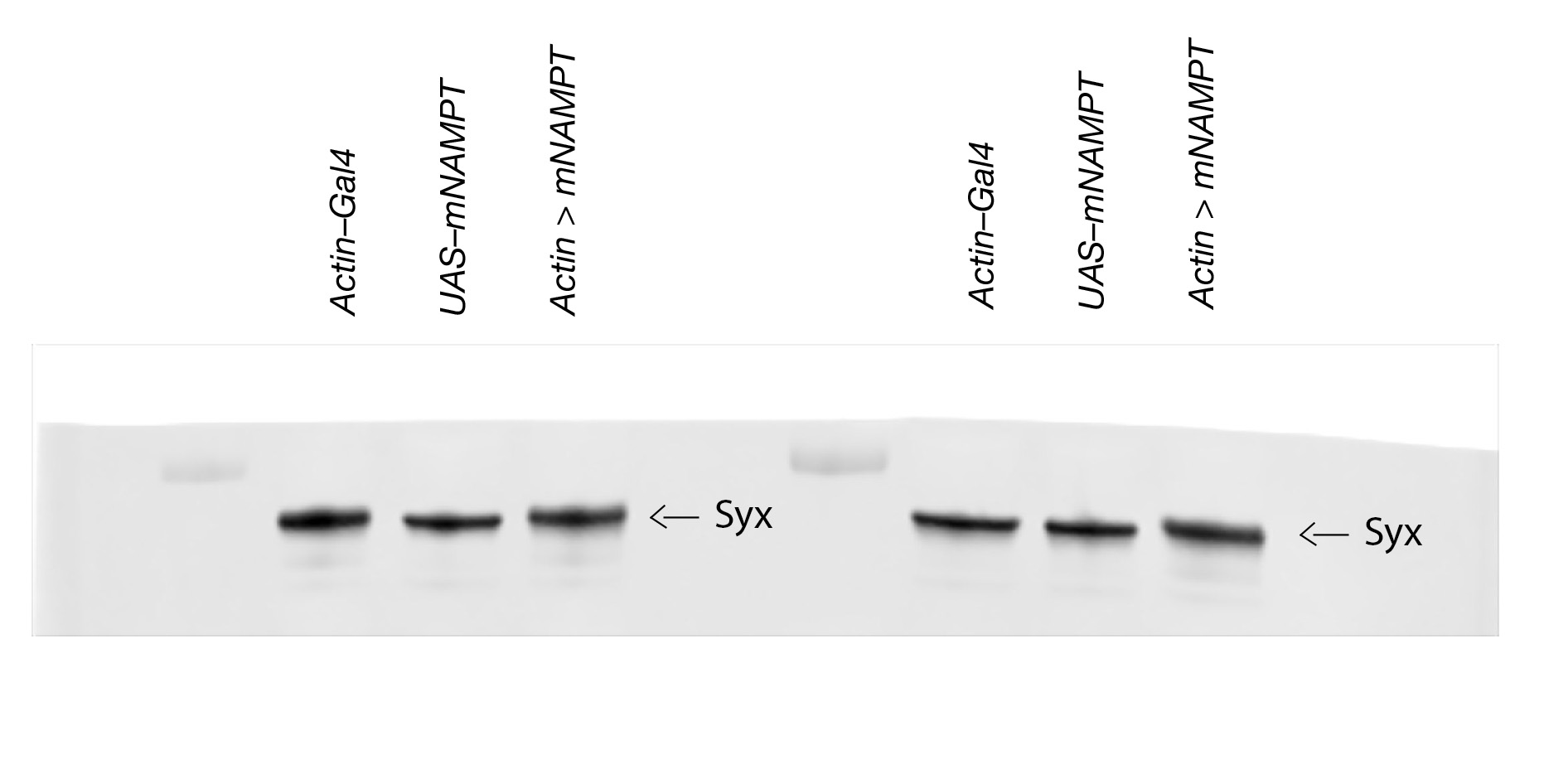

Supplement: Figure 4—source data 1. — (B) Raw unedited Western blots and uncropped blots with relevant bands clearly labeled. (C) Raw data of metabolomic measurement. (E) Raw data of quantified preserved severed axons. (F) Raw data of quantified preserved severed axons. (G) Raw data of quantified preserved severed axons. [file elife-80245-fig4-data1.zip › Figure 4/4B wb/syx + info.jpg]

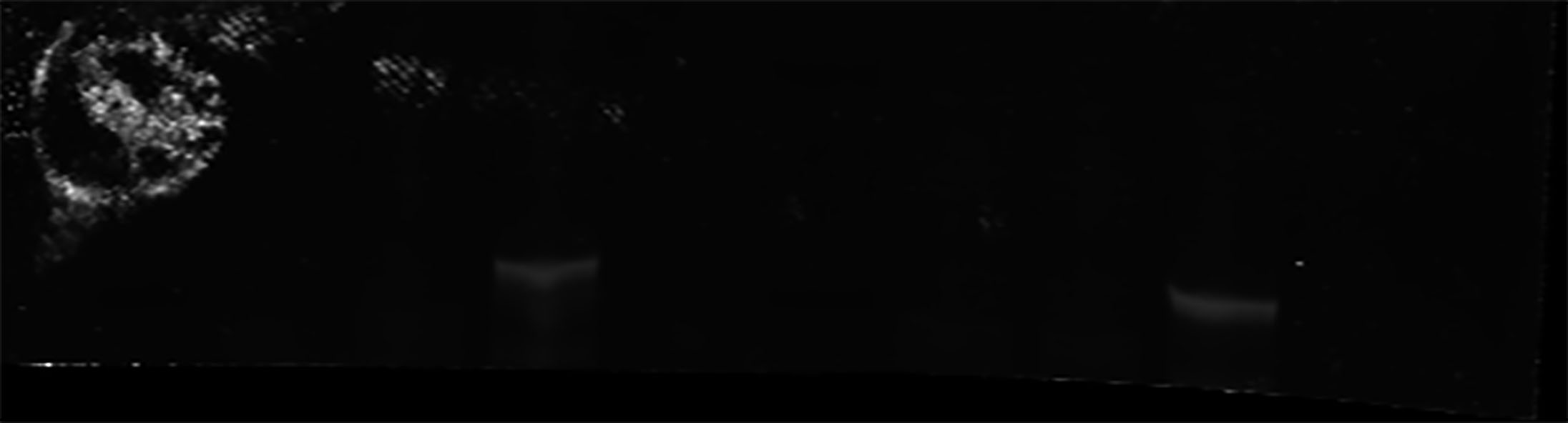

Supplement: Figure 4—source data 1. — (B) Raw unedited Western blots and uncropped blots with relevant bands clearly labeled. (C) Raw data of metabolomic measurement. (E) Raw data of quantified preserved severed axons. (F) Raw data of quantified preserved severed axons. (G) Raw data of quantified preserved severed axons. [file elife-80245-fig4-data1.zip › Figure 4/4B wb/mnampt raw.jpg]

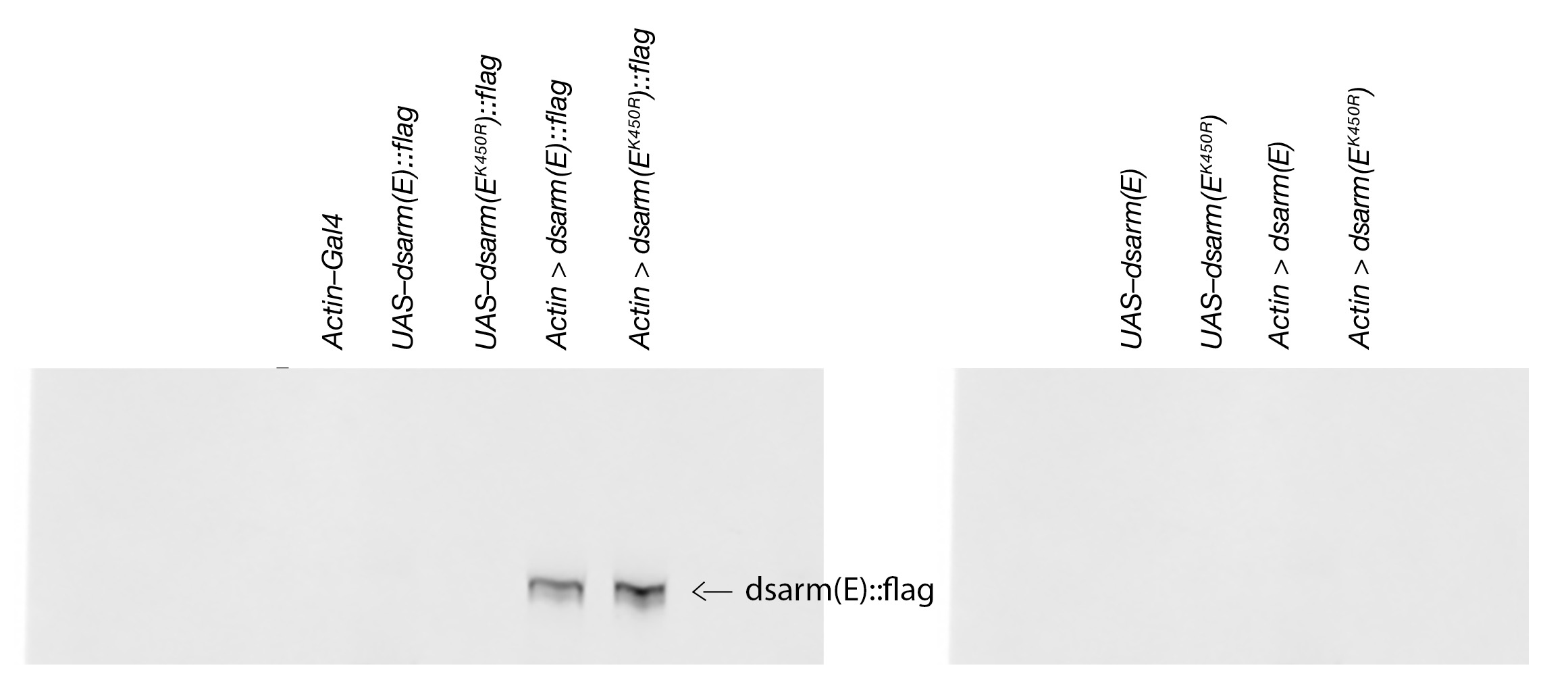

Supplement: Figure 5—source data 1. — (A) Raw unedited Western blots and uncropped blots with relevant bands clearly labeled. (B) Raw unedited Western blots and uncropped blots with relevant bands clearly labeled. (C) Raw data of NADglo assay. (D) Raw unedited Western blots and uncropped blots with relevant bands clearly labeled. (E) Raw data of quantified preserved severed axons. [file elife-80245-fig5-data1.zip › Figure 5/5C Western/flag 1st and 2nd + info.jpg]

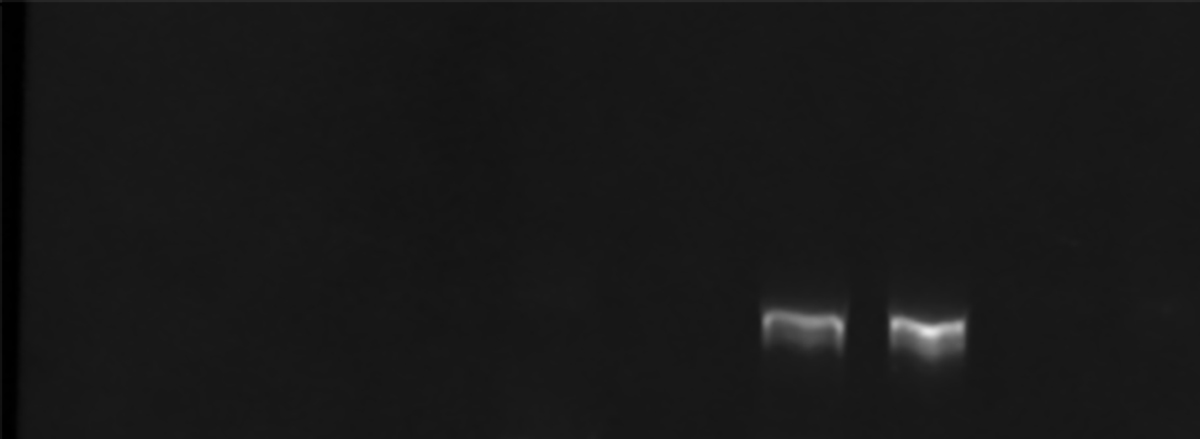

Supplement: Figure 5—source data 1. — (A) Raw unedited Western blots and uncropped blots with relevant bands clearly labeled. (B) Raw unedited Western blots and uncropped blots with relevant bands clearly labeled. (C) Raw data of NADglo assay. (D) Raw unedited Western blots and uncropped blots with relevant bands clearly labeled. (E) Raw data of quantified preserved severed axons. [file elife-80245-fig5-data1.zip › Figure 5/5C Western/flag 1st wb raw.jpg]

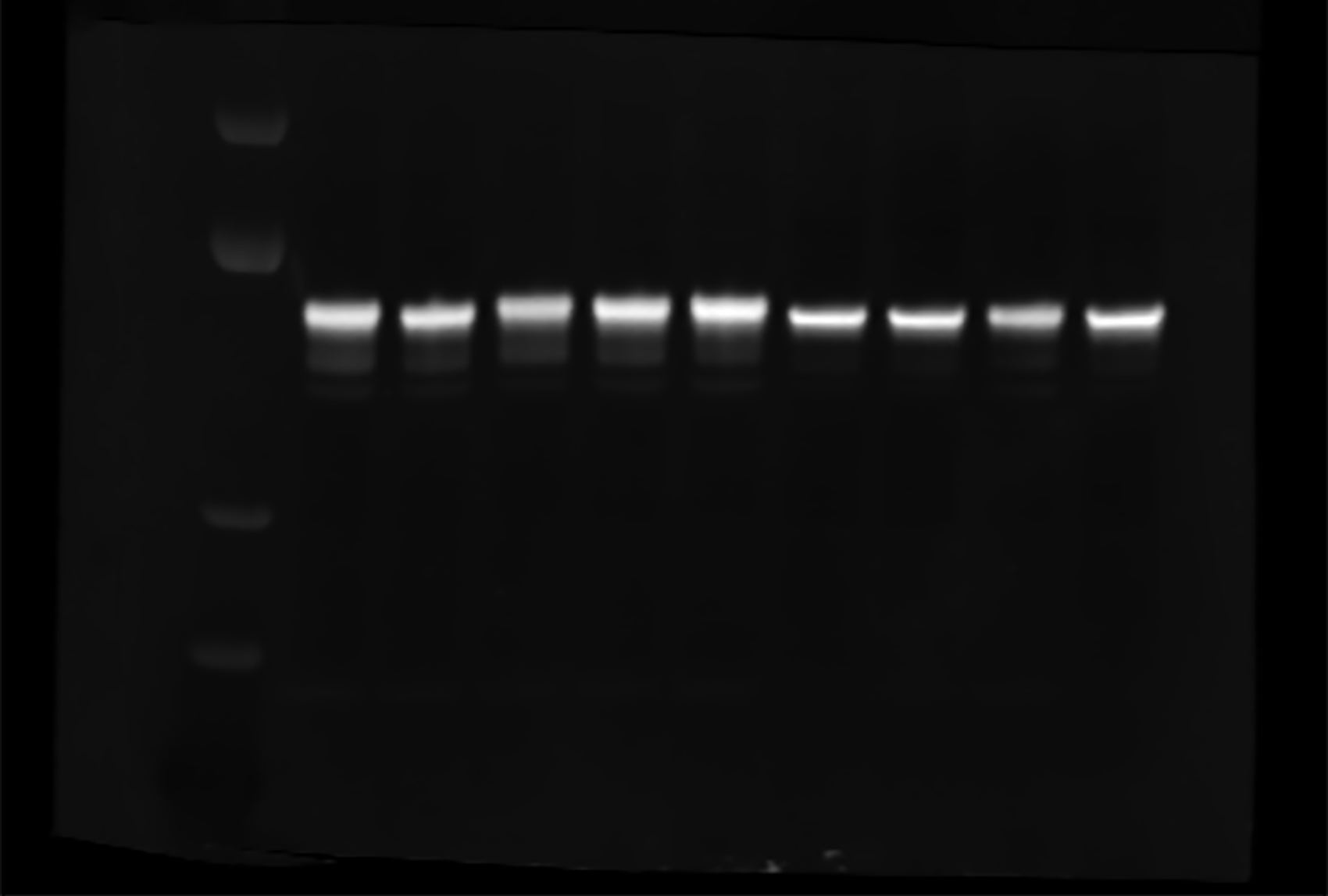

Supplement: Figure 5—source data 1. — (A) Raw unedited Western blots and uncropped blots with relevant bands clearly labeled. (B) Raw unedited Western blots and uncropped blots with relevant bands clearly labeled. (C) Raw data of NADglo assay. (D) Raw unedited Western blots and uncropped blots with relevant bands clearly labeled. (E) Raw data of quantified preserved severed axons. [file elife-80245-fig5-data1.zip › Figure 5/5C Western/syx 1st and 2nd wb raw.tif]

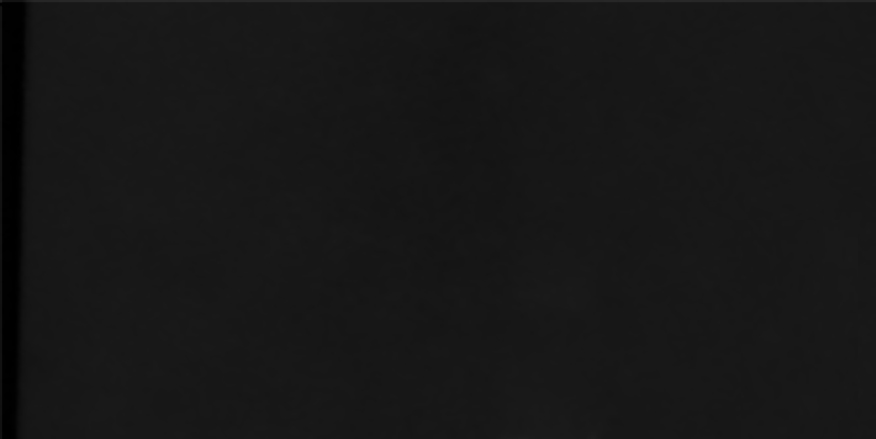

Supplement: Figure 5—source data 1. — (A) Raw unedited Western blots and uncropped blots with relevant bands clearly labeled. (B) Raw unedited Western blots and uncropped blots with relevant bands clearly labeled. (C) Raw data of NADglo assay. (D) Raw unedited Western blots and uncropped blots with relevant bands clearly labeled. (E) Raw data of quantified preserved severed axons. [file elife-80245-fig5-data1.zip › Figure 5/5C Western/flag 2nd wb raw.jpg]

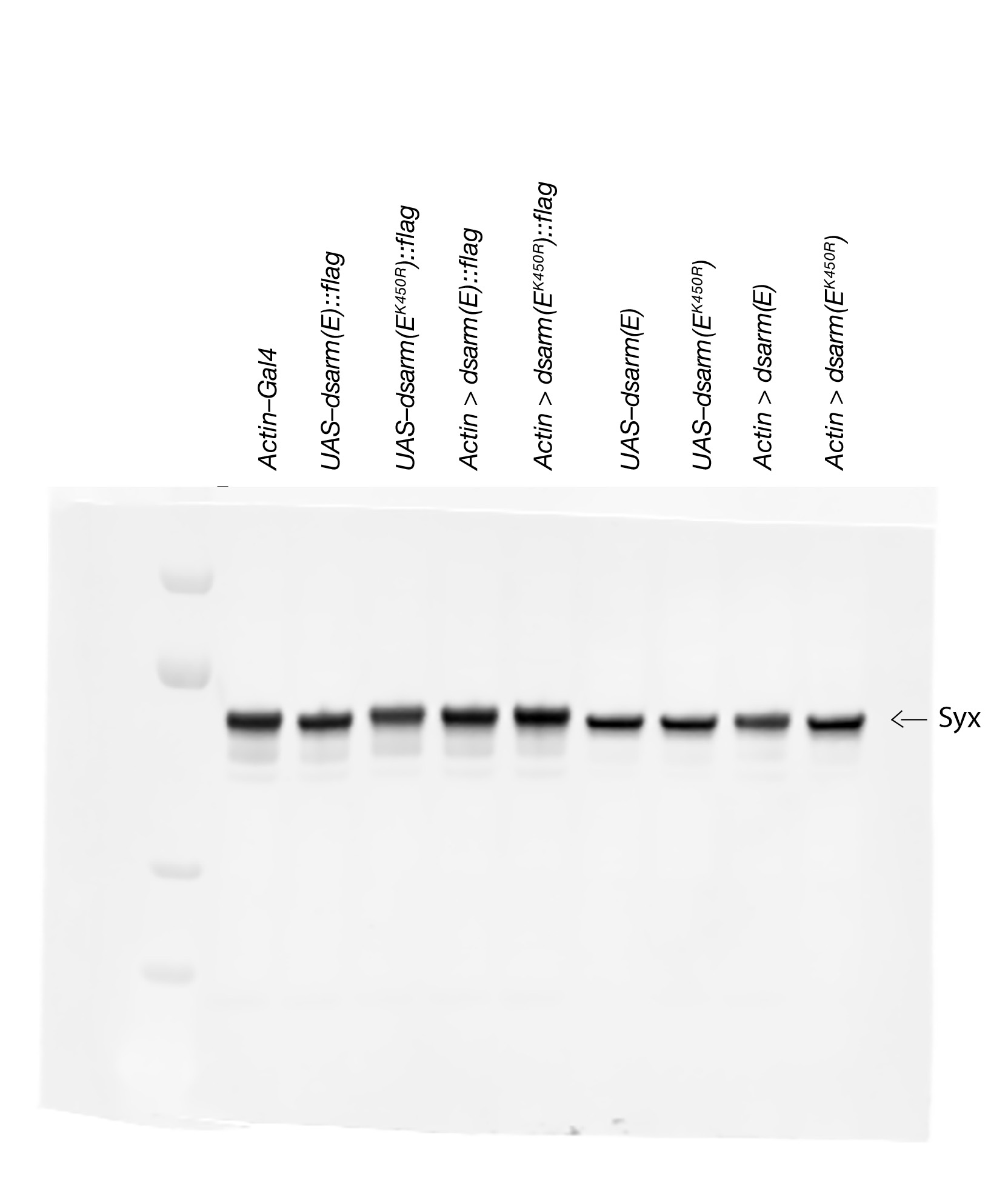

Supplement: Figure 5—source data 1. — (A) Raw unedited Western blots and uncropped blots with relevant bands clearly labeled. (B) Raw unedited Western blots and uncropped blots with relevant bands clearly labeled. (C) Raw data of NADglo assay. (D) Raw unedited Western blots and uncropped blots with relevant bands clearly labeled. (E) Raw data of quantified preserved severed axons. [file elife-80245-fig5-data1.zip › Figure 5/5C Western/syx 1st and 2nd wb + info.jpg]

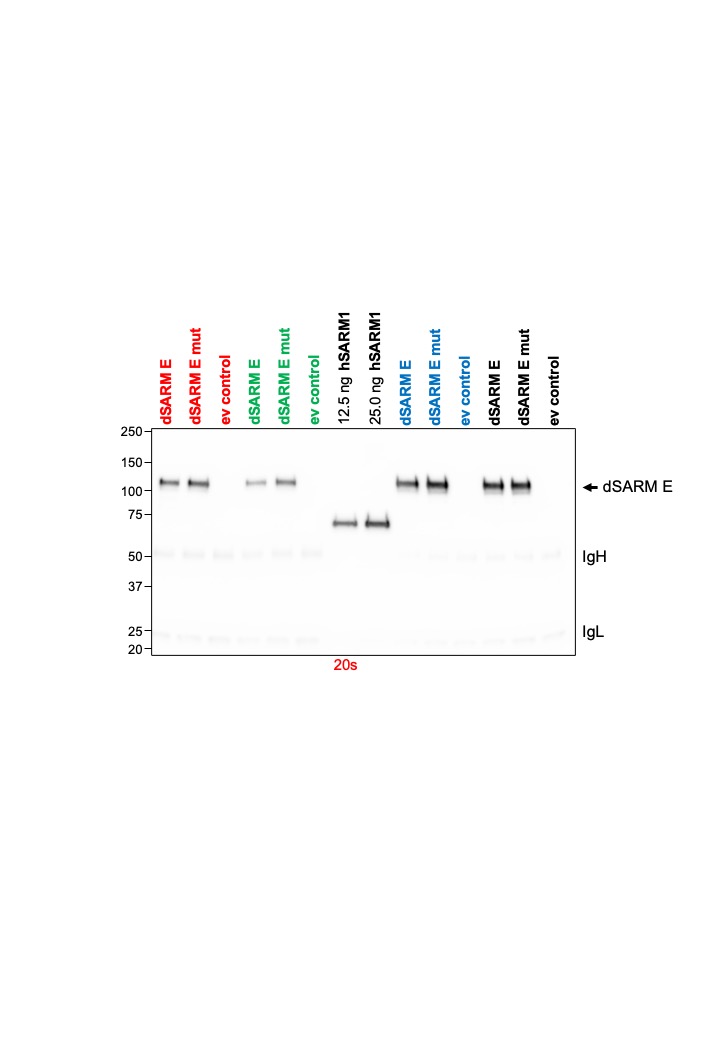

Supplement: Figure 5—source data 1. — (A) Raw unedited Western blots and uncropped blots with relevant bands clearly labeled. (B) Raw unedited Western blots and uncropped blots with relevant bands clearly labeled. (C) Raw data of NADglo assay. (D) Raw unedited Western blots and uncropped blots with relevant bands clearly labeled. (E) Raw data of quantified preserved severed axons. [file elife-80245-fig5-data1.zip › Figure 5/5B Western/flag + info.jpeg]

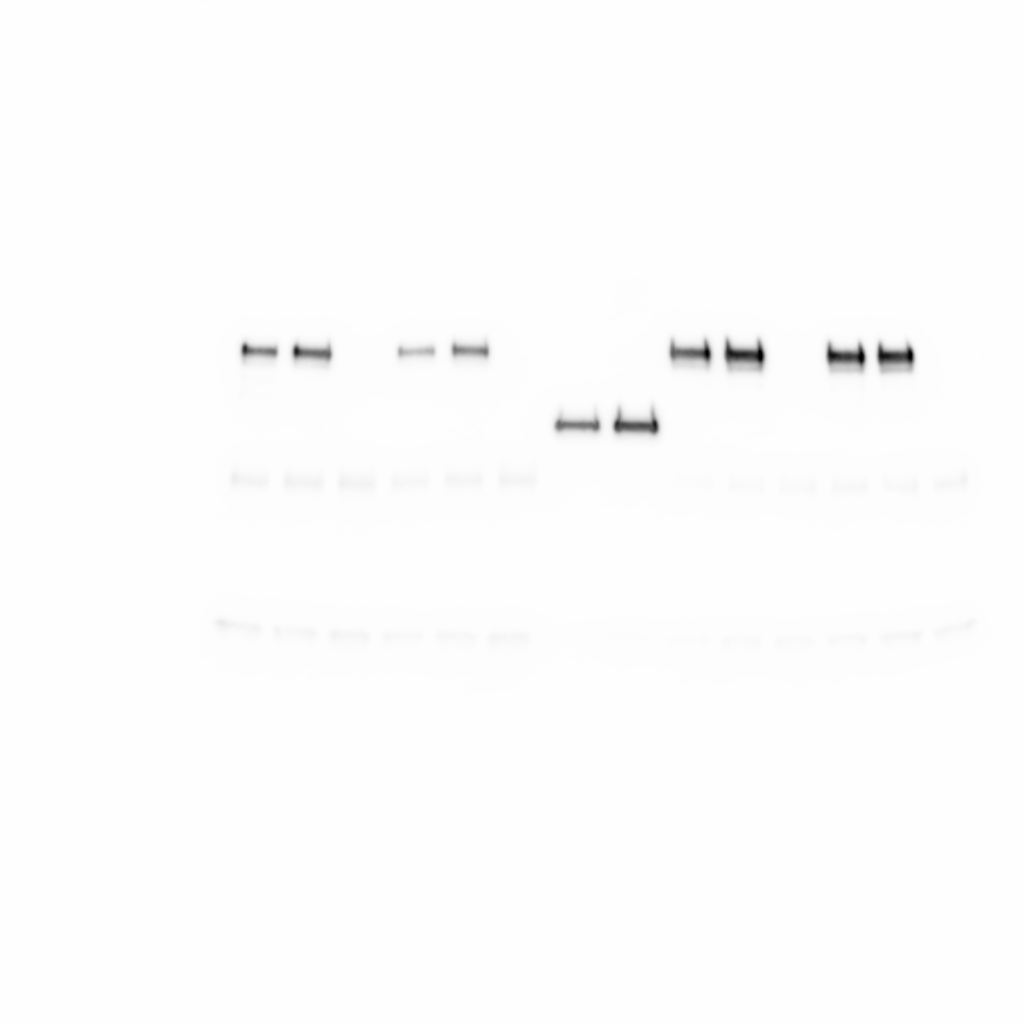

Supplement: Figure 5—source data 1. — (A) Raw unedited Western blots and uncropped blots with relevant bands clearly labeled. (B) Raw unedited Western blots and uncropped blots with relevant bands clearly labeled. (C) Raw data of NADglo assay. (D) Raw unedited Western blots and uncropped blots with relevant bands clearly labeled. (E) Raw data of quantified preserved severed axons. [file elife-80245-fig5-data1.zip › Figure 5/5B Western/flag raw.Tif]

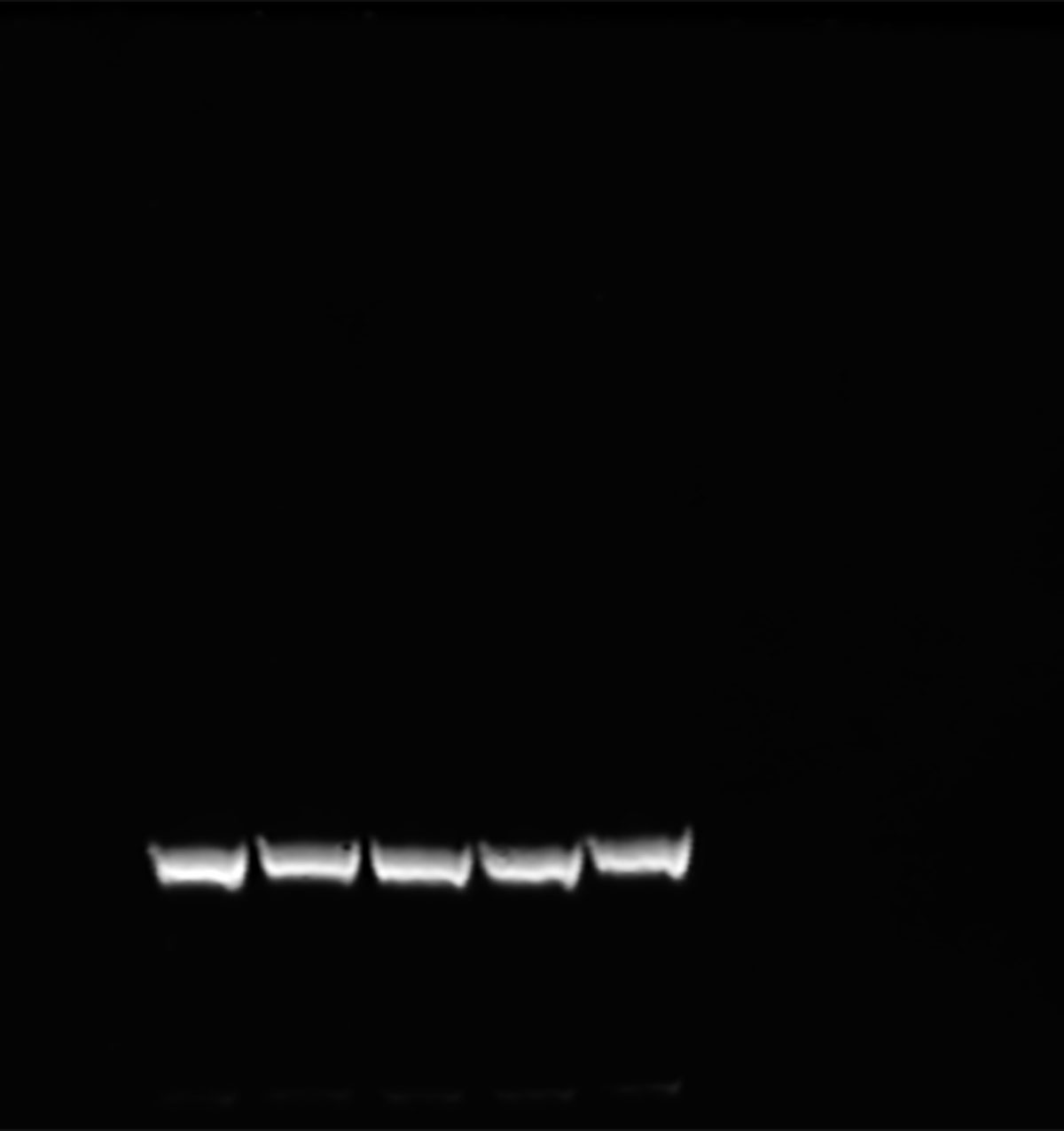

Supplement: Figure 5—source data 1. — (A) Raw unedited Western blots and uncropped blots with relevant bands clearly labeled. (B) Raw unedited Western blots and uncropped blots with relevant bands clearly labeled. (C) Raw data of NADglo assay. (D) Raw unedited Western blots and uncropped blots with relevant bands clearly labeled. (E) Raw data of quantified preserved severed axons. [file elife-80245-fig5-data1.zip › Figure 5/5A Western/syx raw.jpg]

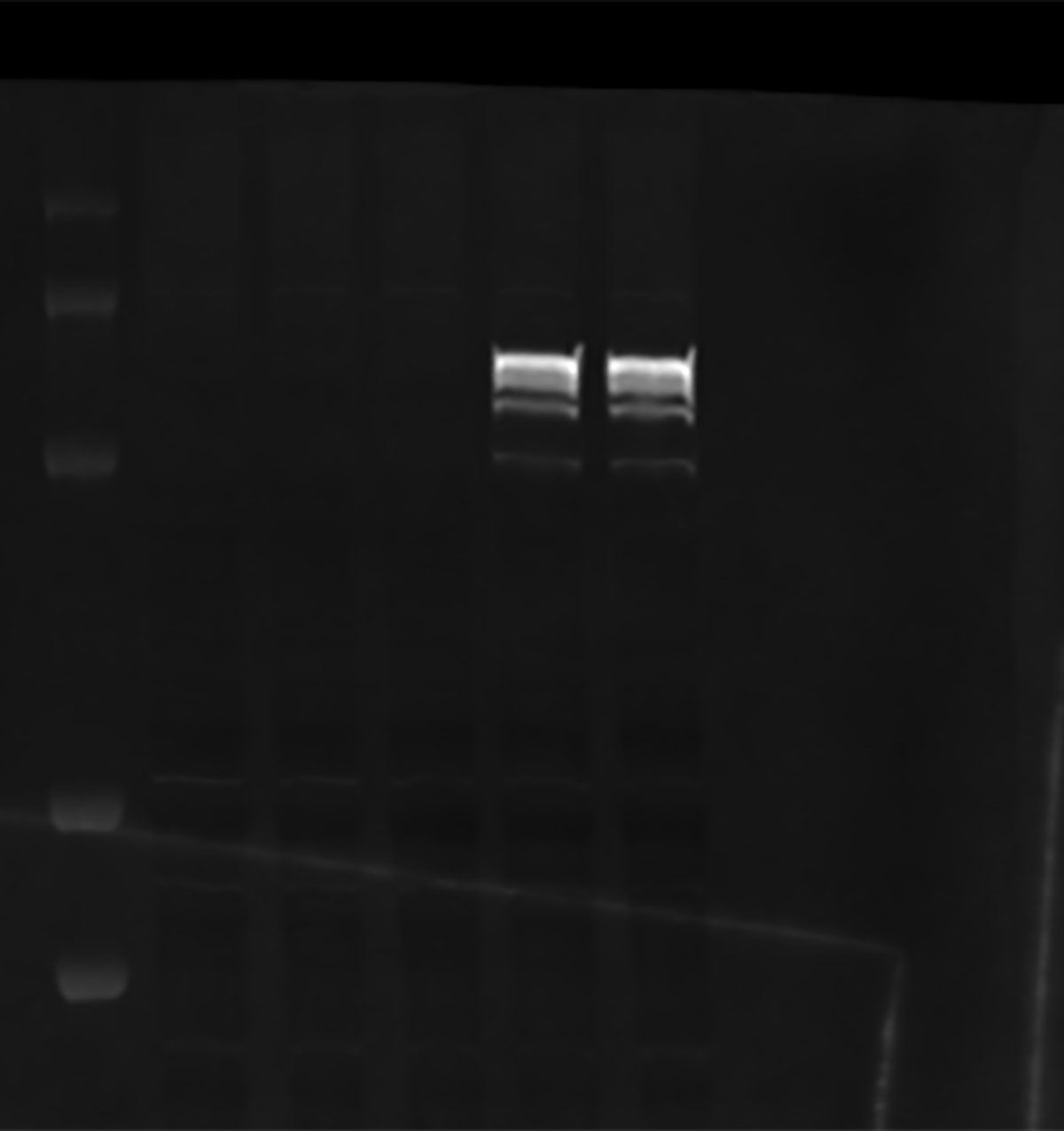

Supplement: Figure 5—source data 1. — (A) Raw unedited Western blots and uncropped blots with relevant bands clearly labeled. (B) Raw unedited Western blots and uncropped blots with relevant bands clearly labeled. (C) Raw data of NADglo assay. (D) Raw unedited Western blots and uncropped blots with relevant bands clearly labeled. (E) Raw data of quantified preserved severed axons. [file elife-80245-fig5-data1.zip › Figure 5/5A Western/flag raw.jpg]

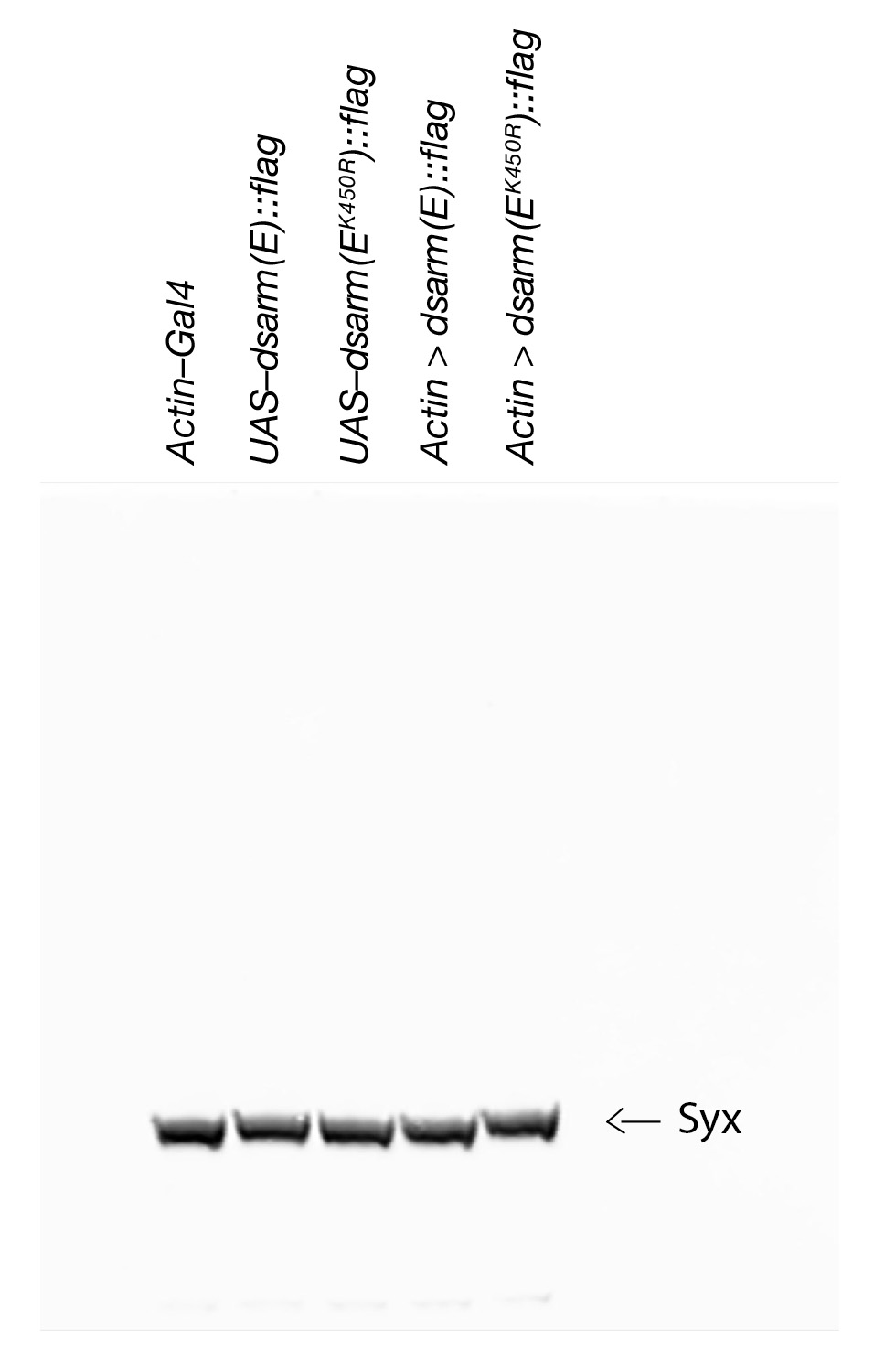

Supplement: Figure 5—source data 1. — (A) Raw unedited Western blots and uncropped blots with relevant bands clearly labeled. (B) Raw unedited Western blots and uncropped blots with relevant bands clearly labeled. (C) Raw data of NADglo assay. (D) Raw unedited Western blots and uncropped blots with relevant bands clearly labeled. (E) Raw data of quantified preserved severed axons. [file elife-80245-fig5-data1.zip › Figure 5/5A Western/syx + info.jpg]

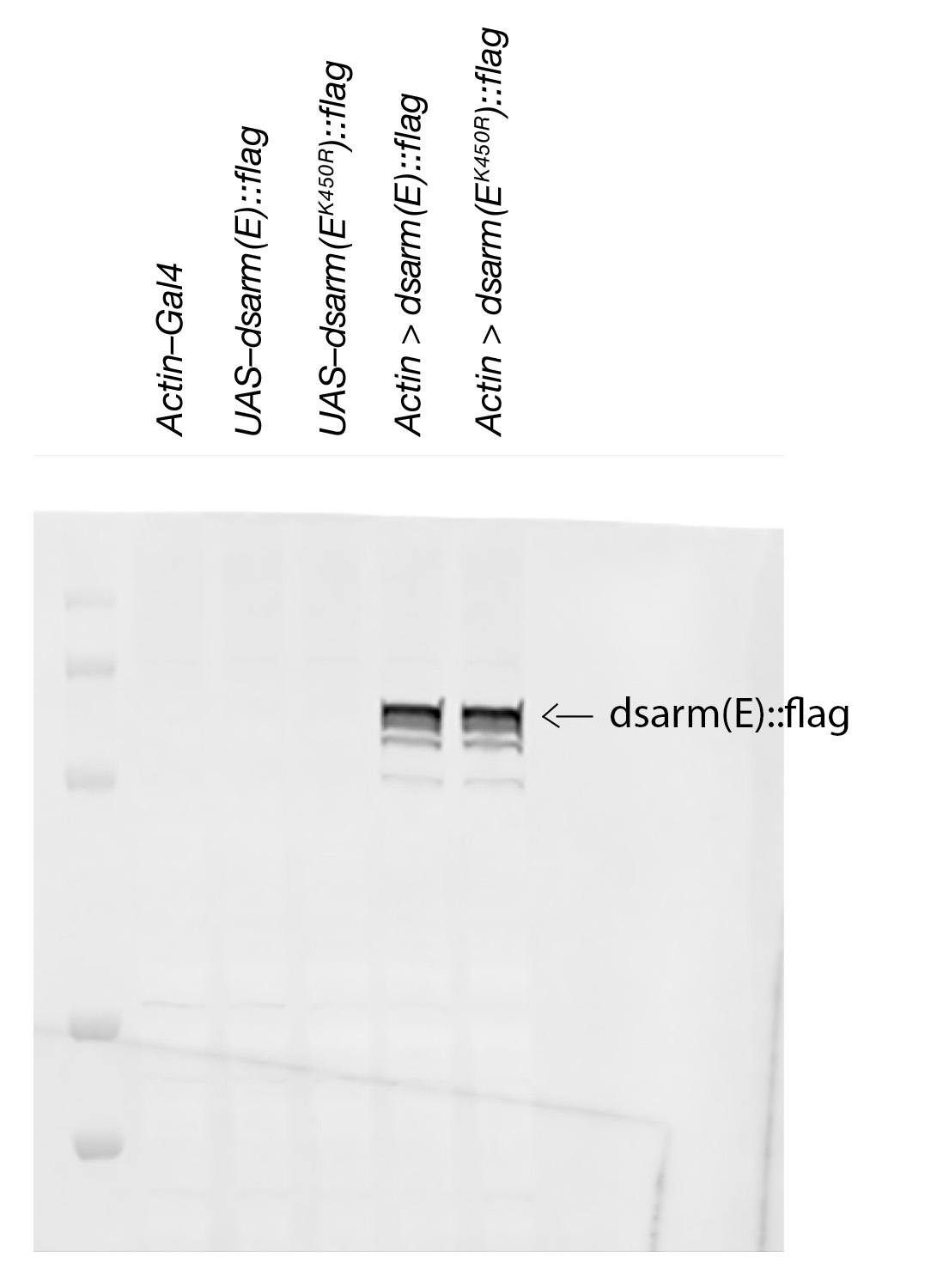

Supplement: Figure 5—source data 1. — (A) Raw unedited Western blots and uncropped blots with relevant bands clearly labeled. (B) Raw unedited Western blots and uncropped blots with relevant bands clearly labeled. (C) Raw data of NADglo assay. (D) Raw unedited Western blots and uncropped blots with relevant bands clearly labeled. (E) Raw data of quantified preserved severed axons. [file elife-80245-fig5-data1.zip › Figure 5/5A Western/flag + info.jpg]

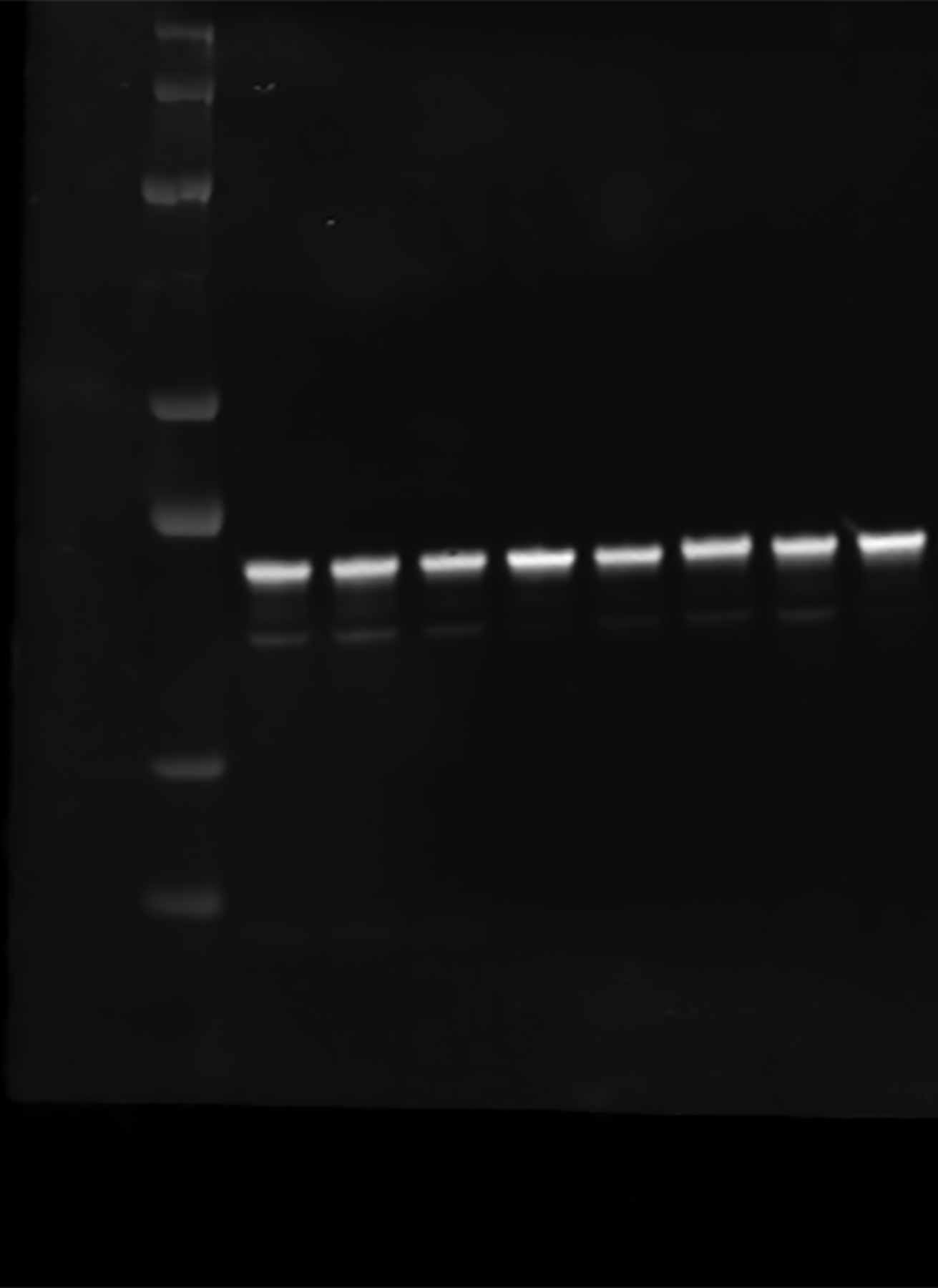

Supplement: Figure 5—figure supplement 1—source data 1. — (A) Raw unedited western blots and uncropped blots with relevant bands clearly labeled. (B) Raw data of quantified preserved severed axons. [file elife-80245-fig5-figsupp1-data1.zip › Figure 5 - figure supplement 1/5 S1A Western/syx raw.jpg]

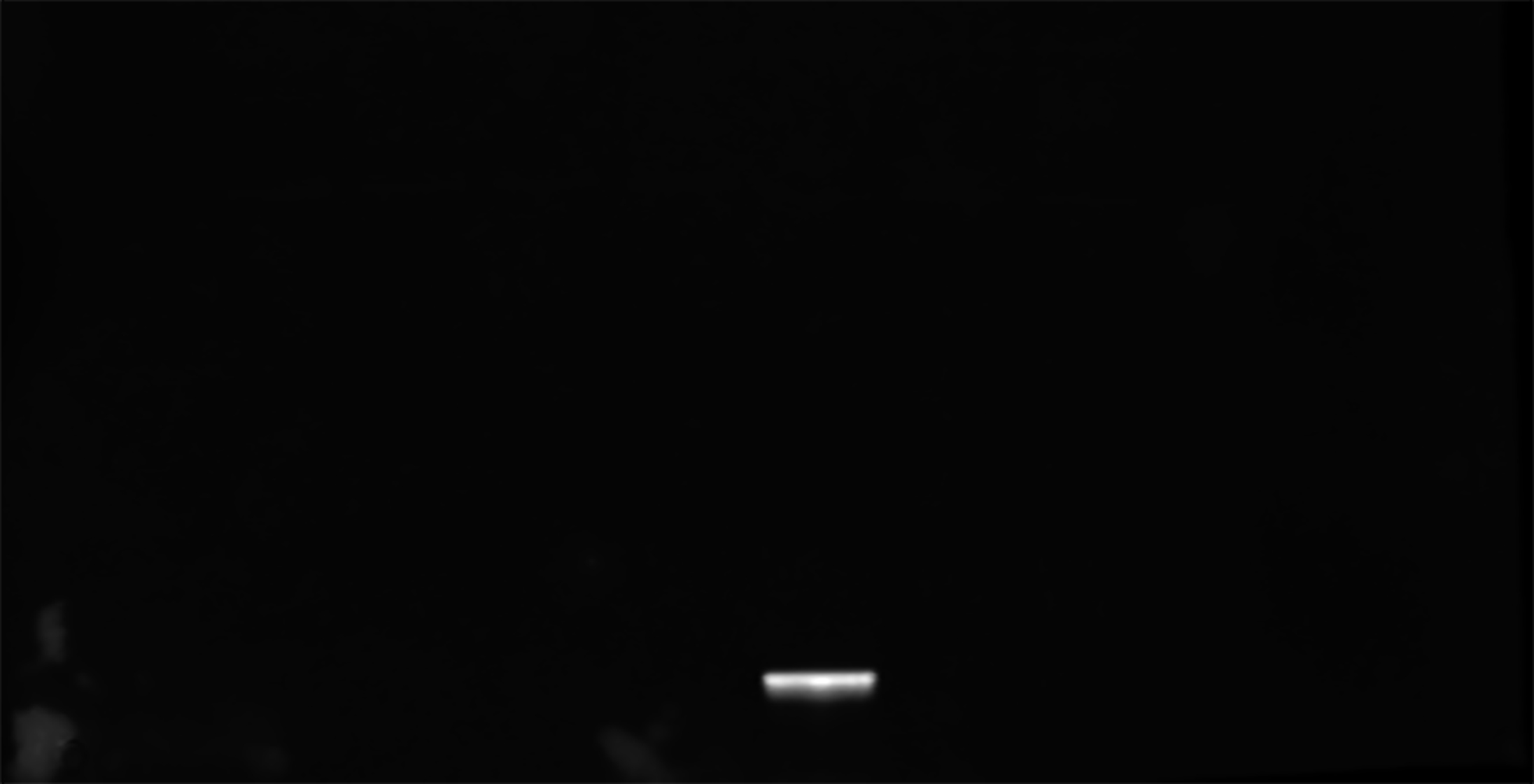

Supplement: Figure 5—figure supplement 1—source data 1. — (A) Raw unedited western blots and uncropped blots with relevant bands clearly labeled. (B) Raw data of quantified preserved severed axons. [file elife-80245-fig5-figsupp1-data1.zip › Figure 5 - figure supplement 1/5 S1A Western/gfp raw.jpg]

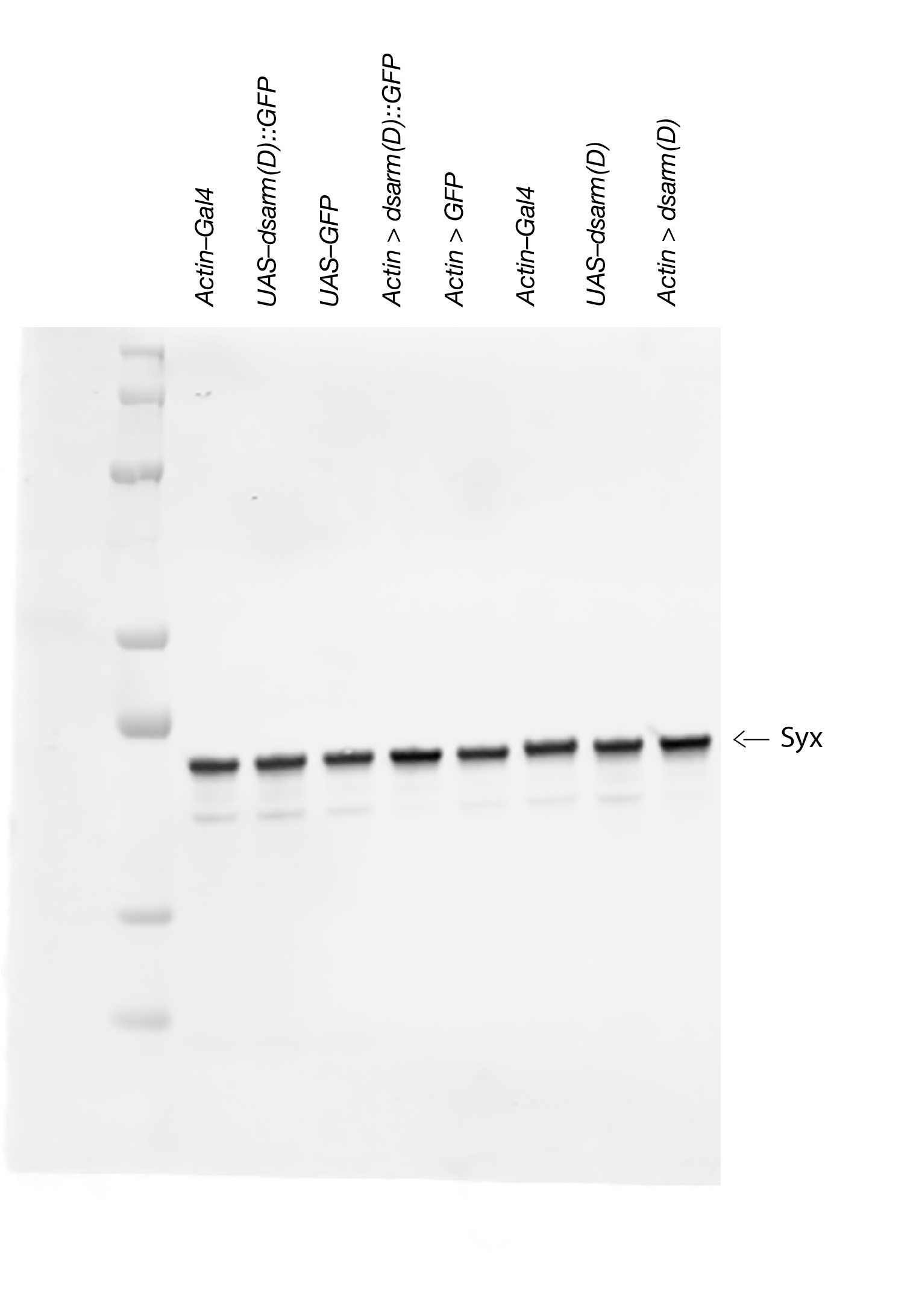

Supplement: Figure 5—figure supplement 1—source data 1. — (A) Raw unedited western blots and uncropped blots with relevant bands clearly labeled. (B) Raw data of quantified preserved severed axons. [file elife-80245-fig5-figsupp1-data1.zip › Figure 5 - figure supplement 1/5 S1A Western/syx + info.jpg]

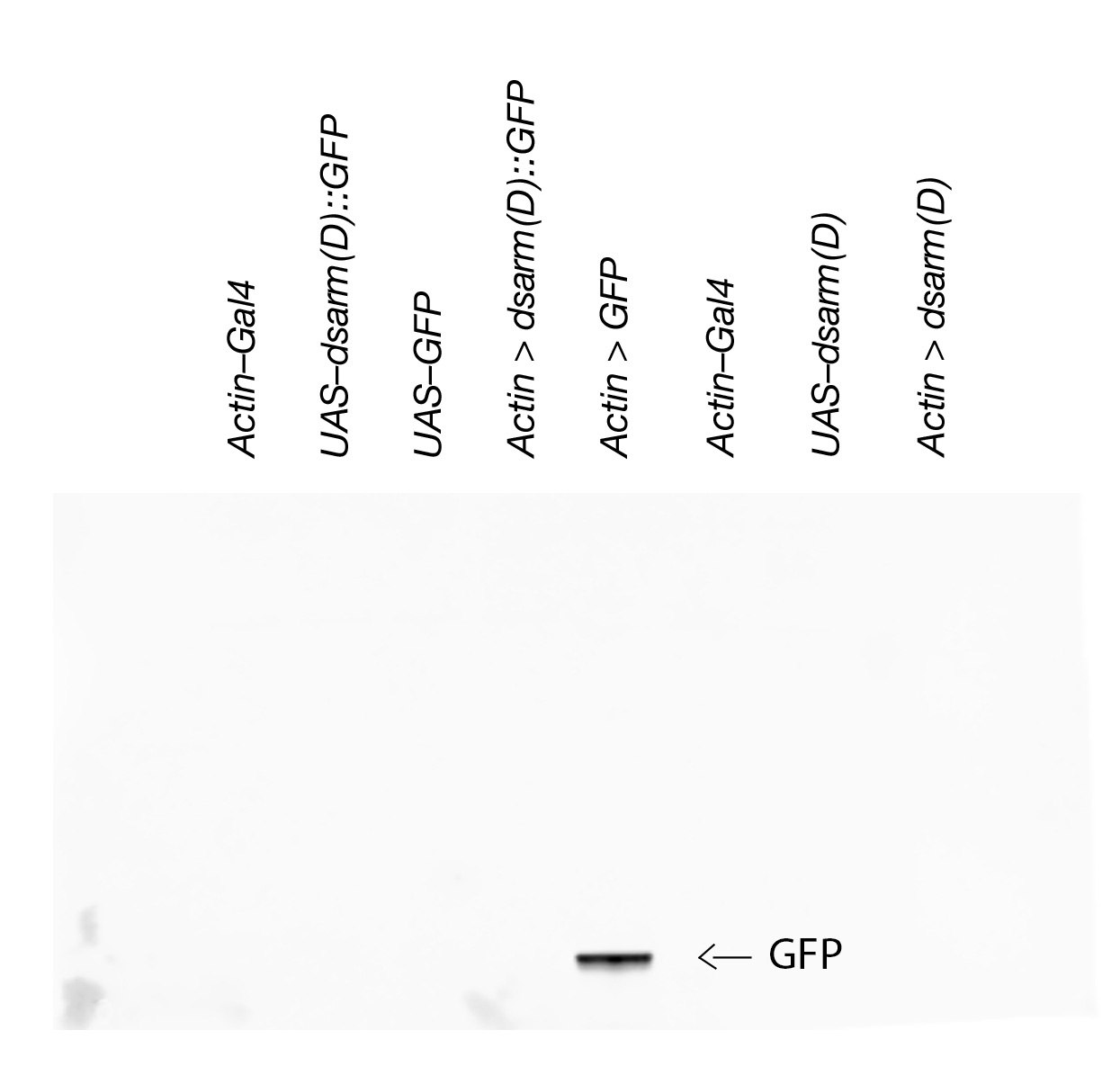

Supplement: Figure 5—figure supplement 1—source data 1. — (A) Raw unedited western blots and uncropped blots with relevant bands clearly labeled. (B) Raw data of quantified preserved severed axons. [file elife-80245-fig5-figsupp1-data1.zip › Figure 5 - figure supplement 1/5 S1A Western/gfp + info.jpg]
